# Goal-Directed Action Is Initially Impaired in a hAPP-J20 Mouse Model of Alzheimer’s Disease

**DOI:** 10.1523/ENEURO.0363-22.2023

**Published:** 2023-02-13

**Authors:** Amolika Dhungana, Serena Becchi, Jessica Leake, Gary Morris, Nesli Avgan, Bernard W. Balleine, Bryce Vissel, Laura A. Bradfield

**Affiliations:** 1 School of Life Sciences, Faculty of Science, University of Technology Sydney, Sydney, New South Wales 2007, Australia; 2 Centre for Neuroscience and Regenerative Medicine, St. Vincent’s Centre for Applied Medical Research, St. Vincent’s Health Network, Sydney, New South Wales 2010, Australia; 3 School of Psychology, University of New South Wales Sydney, Sydney, New South Wales 2052, Australia; 4 Tasmanian School of Medicine, College of Health and Medicine, University of Tasmania, Hobart, Tasmania 7000, Australia; 5 Decision Neuroscience Laboratory, School of Psychology, University of New South Wales Sydney, Sydney, New South Wales 2052, Australia; 6 School of Clinical Medicine, University of New South Wales Medicine & Health, St Vincent’s Healthcare Clinical Campus, Faculty of Medicine and Health, University of New South Wales Sydney, Sydney, New South Wales 2052, Australia

**Keywords:** Alzheimer’s, decision-making, goal-directed action, hAPP-J20 mice, hippocampus, neuroinflammation

## Abstract

Cognitive-behavioral testing in preclinical models of Alzheimer’s disease has failed to capture deficits in goal-directed action control. Here, we provide the first comprehensive investigation of goal-directed action in a transgenic mouse model of Alzheimer’s disease. Specifically, we tested outcome devaluation performance in male and female human amyloid precursor protein (hAPP)-J20 mice. Mice were first trained to press left and right levers for pellet and sucrose outcomes, respectively (counterbalanced), over 4 d. On test, mice were prefed one of the outcomes to satiety and given a choice between levers. Devaluation performance was intact for 36-week-old wild-types of both sexes, who responded more on the valued relative to the devalued lever (Valued > Devalued). By contrast, devaluation was impaired (Valued = Devalued) for J20 mice of both sexes, and for 52-week-old male mice regardless of genotype. After additional lever press training (i.e., 8-d lever pressing in total), devaluation was intact for all mice, demonstrating that the initial deficits were not a result of a nonspecific impairment in reward processing, depression, or locomotor activity in J20 or aging mice. Follow-up analyses revealed that microglial expression in the dorsal CA1 region of the hippocampus was associated with poorer outcome devaluation performance on initial, but not later tests. Together, these data demonstrate that goal-directed action is initially impaired in J20 mice of both sexes and in aging male mice regardless of genotype, and that this impairment is related to neuroinflammation in the dorsal CA1 hippocampal region.

## Significance Statement

Treatments for Alzheimer’s disease trialed in preclinical animal models have repeatedly failed to translate to the clinic. One potential reason for this could be that the cognitive-behavioral assays used in such models have been limited to one aspect of Alzheimer impairment: visuospatial memory. Here, we demonstrate that male and female mice belonging to the transgenic human amyloid precursor protein (hAPP)-J20 mouse model also display consistent deficits in the initial acquisition of goal-directed action. This study therefore represents an important step toward the broader capture of Alzheimer-like cognitive deficits at a preclinical level which could improve translatability when used to more comprehensively test treatment efficacy prior to clinical trials.

## Introduction

Alzheimer’s disease causes impairments in memory, cognition, and behavior (Kidd, 2008; Wenk, 2003) and its neuropathological features include neuronal loss, synapse loss, neuroinflammation, tau hyperphosphorylation leading to neurofibrillary tangles (Delacourte and Defossez, 1986), and amyloid-β (Aβ) aggregation into plaques (Glenner and Wong, 1984; Trejo-Lopez et al., 2022). Historically, it is this latter feature that has been considered its most central characteristic (Bharadwaj et al., 2009) yet numerous treatments that have successfully cleared Aβ plaques and produced cognitive-behavioral recovery in rodent models have failed to produce similar recovery in humans (Cummings et al., 2014; Hung and Fu, 2017). Better approaches to translatability are clearly needed. One facet of rodent models that could be straightforwardly improved are the behavioral and cognitive tests used to determine how well such models replicate the symptoms of Alzheimer’s disease.

According to the National Institute of Ageing (NIA), an individual will only be diagnosed with Alzheimer’s if their symptoms of memory loss and visual/spatial problems are “significant enough to impair a person’s ability to function independently,” yet rodent models routinely test locomotor, spatial, or memory deficits without any regard to how these issues might translate into functional outcomes. Goal-directed action control is crucial to independent functioning and its impairment in individuals with Alzheimer’s can be devastating, as it prevents individuals from reaching their goals and from making effective decisions (Brown and Pluck, 2000; Satler et al., 2017). Therefore, it was the aim of the current study to investigate goal-directed action control in a preclinical, human amyloid precursor protein (hAPP)-J20 mouse model of Alzheimer’s disease for the first time. Following the detection of goal-directed deficits, we further set out to characterize the putative brain mechanisms. Ultimately, our hope is that these tests could be more widely employed in addition to currently-used tests (e.g., Barne’s maze, context fear conditioning) to better capture the broad scope of symptoms experienced by patients at a preclinical level. This would increase translatability by ensuring that treatments target multiple aspects of cognitive dysfunction, not simply visuospatial memory.

Goal-directed action is defined as action motivated by a) the current value of the outcome and b) the contingency between the action and the outcome (Balleine and Dickinson, 1998). Outcome devaluation provides a test of these criteria. The organism is typically trained to perform two actions (e.g., left and right lever press) for two outcomes (e.g., pellets and sucrose), the value of one of which is subsequently devalued, often by being fed to satiety or paired with illness. When tested in the absence of the outcomes, the organism that selects the action associated with the still valued outcome is acting under goal-directed control because they are responding in accordance with both the outcome’s value (value criterion) and their memory of which action earned which outcome (contingency criterion). Outcome devaluation has been translated to humans and tested in individuals with several different diseases and disorders (Wong et al., 2018; Morris et al., 2020), where it has been linked to functional outcomes (Alvares et al., 2014; Sjoerds et al., 2016; Byrne et al., 2019). For these reasons, we chose to apply this test to evaluate goal-directed action using the hAPP-J20 mouse model of Alzheimer’s disease generated by Mucke et al. (2000).

We chose this transgenic model for two reasons. First, because this line expresses human APP (hAPP) bearing two mutations; the Swedish (K595N) and Indiana (M596L) mutation, it leads to increased Aβ plaque load, neuronal loss, and neuroinflammation relative to wild-types; neuropathological features that have been extensively characterized in the dorsal CA1 region of the hippocampus (Yiu et al., 2011; Wright et al., 2013). This is of interest because we recently demonstrated that inactivating this same brain region caused an initial deficit in devaluation performance that could be overcome with additional training (Bradfield et al., 2020). Therefore, we hypothesized that the damage suffered to the dorsal CA1 in J20 mice would likewise cause an initial impairment in devaluation that could be overcome with additional training. Second, J20 mice have demonstrated clear cognitive/behavioral deficits across several of the “traditional” tests mentioned above, including increased locomotor activity (Cheng et al., 2007) and impaired spatial memory (Cissé et al., 2011; Wright et al., 2013; Yoshikawa et al., 2018; Flores et al., 2022), suggesting that these mice are behaviorally abnormal and thus an ideal initial candidate for identifying impairments in goal-directed action. Following behavioral testing we conducted a follow-up analysis to identify the potential mechanisms of the observed behavioral impairments. Specifically, we quantified immunohistochemical markers of Aβ deposition (Amylo-Glo), and of putative neuroinflammation such as ionized calcium binding adaptor molecule 1 (IBA1) and glial fibrillary acidic protein (GFAP) in the dorsal CA1 region of a subset of mice, which we then compared between groups and correlated with a measure of devaluation performance from both tests.

## Materials and Methods

### Subjects

A total of 58 hemizygous transgenic (hAPP-J20) and nontransgenic mice (WT) from the J20 line, which express hAPP containing both the Swedish and Indiana mutations under a PDGF-β chain promoter, were used for this study. Four mice were excluded for failing to learn to lever press (i.e., for performing no more than a single lever press before the 4-d test), and 1 was found dead, possibly as a result of a seizure which this mouse line is prone to (Palop et al., 2007). Thirty-nine of the remaining mice were males, 15 of which were ∼36 weeks at the start of behavioral testing and 24 were ∼52 weeks. All nineteen females were ∼36 weeks at the start of testing. Regrettably, no older females were available, because of the historical bias against breeding females that current breeding protocols in our laboratories is aiming to address. Mice were housed in sibling groups of two to five, separated by sex. We have reported results for female mice separately below because slightly different experimental parameters were used for male and females, due to of female mice exhibiting substantially lower lever press rates.

Mice were bred and housed at Australian BioResources located in Mossvale, New South Wales, Australia until their issue to Garvan Institute, New South Wales, Australia. As litters reached the desired age range at different times, behavioral testing occurred in a staggered fashion (i.e., the age at which animals in the same group were in the age range for testing did not always overlap). However, all experimental and nonexperimental (e.g., housing) variables were kept as consistent as possible to minimize any potential impacts of variables other than the target variables influencing performance. Upon arrival, mice weighed between 15–25 g (females) and 20–40 g (males).

Mice were maintained at a 12-h 7 A.M.–7 P.M. light/dark cycle and all experimentation was conducted in the light portion of the cycle. Mice were given *ad libitum* access to food and water until experimentation began. Three days before behavioral training, the mice underwent dietary restrictions whereby each mouse was given 1–2 g of chow per day, and unlimited access to water. These food restrictions were upheld for the duration of the experiment. During this time, mice were handled and weighed every second day to maintain their weight at >80% of their baseline body weight. All experiments were conducted in accordance with the procedures of the Garvan Institute of Medical Research ethics committee.

### Apparatus

Training and experimentation was conducted in six identical operant chambers (Med Associates) equipped with a pump that delivered a 20% sucrose solution and a pellet dispenser that delivered a single grain of pellet (Able Scientific) into the magazine (i.e., food receptacle) located in the middle of the side wall. The chamber also came equipped with two retractable levers, placed on either side of the magazine, a house light for illumination that was situated opposite the magazine, and a house fan which provided constant ∼70-dB background noise. MED-PC software controlled the insertion of levers, delivery of pellet and sucrose outcomes, and recorded the number of lever presses and magazine entries.

### Behavioral procedures

#### Magazine training

Mice first received two sessions of magazine training over 2 d. The start of the session was signaled by illumination of the house light. During the session, sucrose and pellet outcomes were delivered to the magazine at random intervals around a mean of 60 s (i.e., on a random time 60 schedule). The session terminated after 30 min or after 20 of each outcome (40 outcomes in total) had been delivered, whichever came first. Levers were not extended during magazine training.

#### Lever press training, days 1–4

One day after magazine training finished, mice were trained to lever press for 4 d. Each lever press training session lasted for 50 min and consisted of two 10 min periods on each lever (i.e., 4 × 10 min sessions in total) separated by 2.5 min time-out period in which the levers were retracted, and the house light switched off. Lever press periods terminated early if 20 outcomes were earned such that mice could earn a maximum of 40 pellets and 40 deliveries of sucrose solution per session. Contingencies were counterbalanced so that half the animals in each group received left lever-pellets, right lever sucrose, and the remaining half received the opposite arrangement.

For males, the first day of lever press training was continually reinforced (CRF; i.e., each lever press was rewarded with an outcome). They were then shifted to a random ratio (RR)5 schedule for the next 2 d (i.e., each lever earned an outcome with a probability of 0.2), then to a RR10 schedule (i.e., each lever earned an outcome with a probability of 0.1). Female mice were trained on CRF schedules for 2 d, then moved onto 1 d of RR5 and then 1 d of RR10. There was some variability in this schedule according to individual press rates (i.e., slower pressers were kept on richer reward schedules for longer).

#### Outcome devaluation, 4-d test

The first round of devaluation testing that occurred after 4 d of lever press training is referred to as the “4-d test.” For these tests, mice were each placed in an empty Perspex vivarium box and were given *ad libitum* access to either pellets or sucrose (counterbalanced) for 1 h to induce specific satiety, reducing its value relative to the other outcome (Balleine and Dickinson, 1998). Immediately following devaluation, male mice were placed back in the operant chambers for 10 min, whereas female mice were placed in the operant chambers for 5 min. Both the levers were extended, but neither outcome was actually delivered (i.e., test was conducted in extinction). On day 2 of devaluation testing, 24 h after the first, animals were prefed the alternative food source (i.e., if they were prefed pellets on day 1, they received sucrose, and vice versa) and were again tested in extinction for 10 min (males) or 5 min (females). Test results are reported as averaged across these 2 d of testing.

#### Lever press training, days 5–8

One day after the 4-d devaluation test, all mice received four more days of lever press training. These training sessions took place as described above. All mice, regardless of sex, were trained on CRF, RR5, RR10, RR10 over days 5–8.

#### Outcome devaluation, 8-d test

Mice were again subject to outcome devaluation testing after eight total days of lever press training, in what we referred to as the “8-d test.” This test was conducted identically to the 4-d test, described above.

#### Outcome-selective reinstatement

Outcome-selective reinstatement sessions began with 30 min of extinction, during which the house light was turned on and both levers extended and lever presses recorded, but no outcomes were delivered. During reinstatement, mice received four reinstatement trials separated by 4 min each. Each reinstatement trial consisted of a single free delivery of either the sucrose solution or the grain pellet. All rats received the same trial order: sucrose, pellet, pellet, sucrose. Responding was measured during the 2-min periods immediately before (Pre) and after (Post) each delivery.

### Tissue collection

After behavioral testing was finished, each mouse was injected intraperitoneally with a combination of ketamine (2 mg/ml) and xylazine (8 mg/ml), according to their individual weight to deeply anesthetize them. Once anesthetized, the mice were cut open from their abdominal region until the incision reached the ribcage, and the heart was exposed. The apex of the heart was punctured with a needle and a tiny incision was made on the right atrium, allowing the excess liquid to drain out. First, the needle delivered a saline solution which pumped through the blood vessels of the mice to flush out the blood. Following this, the mice were transcardially perfused with 4% paraformaldehyde (PFA). Next, the brains were harvested and postfixed for 24 h in 4% PFA to prevent it from decaying by terminating any biological reactions. The brains were then transferred to a 30% sucrose solution and finally sectioned coronally (40 μm) using a cryostat (Leica Biosystems). The sectioned slices were immediately immersed in cryoprotectant solution and stored at −20°C.

### Immunohistochemistry

Five representative anterior-posterior sections (coordinates of the sections ranged from bregma −1.90 mm to bregma −2.2 mm) from the CA1 region of the dorsal hippocampus were selected for each mouse for IBA1/Amylo-Glo staining procedures to stain for microglia and amyloid plaques, respectively. Additionally, five separate sections from the same region were selected for staining with both GFAP and Amylo-Glo to detect astrocyte expression and amyloid plaques, respectively. Because of a freezer malfunction, several of our brain samples were either lost or damaged to a point that we were unable to image them. Nevertheless, we were able to include the majority of samples in our analysis. All sections were first washed three times (10 min per wash) in sterile phosphate buffered saline (PBS) (pH 7.2) to remove any exogenous substances. The sections were then incubated in a blocking solution comprising of 3% bovine serum albumin (BSA) +0.25% Triton X-100 in 1× PBS for 1 h to permeabilize tissue and block any nonspecific binding.

#### Ionized calcium binding adaptor molecule (IBA1)

Following permeabilization treatments and three more 10-min washes in PBS, sections were first incubated in the primary antibody (1:1000 rabbit-IBA1, Abcam) diluted in the same blocking solution described above for 72 h at 4°C. Next, the sections were washed three more times in PBS followed by incubation in the secondary antibody (anti-rabbit Alexa Fluor-488, Abcam) diluted in blocking solution (1:250) and preserved overnight at 4°C. The sections were washed in PBS for a final time (3 × 10 min) before being stained by Amylo-Glo as per the procedure detailed below.

#### Glial fibrillary acidic protein (GFAP)

The procedure for GFAP staining was identical to that described above as above for IBA1 except that the primary antibody was anti-GFAP (goat-GFAP, Abcam) diluted at 1:300, and the secondary antibody was Alexa Fluor-488 (anti-goat Alexa-Fluor 488, Abcam) diluted at 1:1000. The sections were washed in PBS for a final time (3 × 10 min) before being stained by Amylo-Glo.

#### Amylo-Glo

Amylo-Glo was used to quantify the number of amyloid plaques in the brain and was used by diluting at 1:500 with 0.9% saline solution. After the sections were stained for IBA1 and GFAP, they were put into the Amylo-Glo solution for 1 h. Sections were then mounted on SuperFrost-plus slides and coverslipped with Vectashield.

### Cell count quantifications of microglia, astrocytes, and amyloid plaques

Selected CA1 sections that were immuno-stained for IBA1 and Amylo Glo, or GFAP and Amylo Glo using the same methods described previously were analyzed under an Axio Imager.Z2 fluorescent microscope (Zeiss), and images of the CA1 region of the hippocampus of both right and left hemispheres were obtained. These images were then analyzed and cells or plaques counted on ImageJ as follows: the image was adjusted to 8-bit and the background subtracted. The threshold for contrast and brightness was adjusted for all images until consistent between images. The size of the cells that ImageJ identified as IBA1 were set between 60 and 600 and set at 80–2000 for GFAP. The integrated density of those cells was measured.

ImageJ counted each cell between our parameters and presented it as a “count.” The area of the CA1 being counted was 19.6 mm^2^, hence, these raw counts were divided by 19.6 to give us counts per mm^2^.

### Intensity quantifications of microglia, astrocytes, and amyloid plaques

The stained and mounted sections were analyzed under a confocal laser scanning microscope (Zeiss-LSM-7110 CLSM, Carl Zeiss) with a 20× optical magnification, resolution 1024 × 1024 pixels and a Kaplan filter (three average scans), centered in the acquisition area. Laser intensity, PMT voltage and offset were maintained constant in all acquisition of the same double immunofluorescence experiment. Images of the region of interest (ROI) of 635.9 × 635.9 mm of the CA1 region of in the hippocampus around bregma −1.9 mm (Paxinos and Franklin, 2001; of both left and right hemispheres) were obtained. Raw 16-bit images were then analyzed using ImageJ software (MacBiophotonics upgrade v. 1.43u, Wayne Rasband, National Institutes of Health). For each marker a mean gray value was obtained from two to five sections and averaged for each animal. All measurements were quantified by a researcher who was blind to the group identity. The representative images were chosen and imported to ImageJ where the double stained IBA1/Amylo-Glo or GFAP/Amylo-Glo were counted. An average of five sections were counted.

Amyloid plaque quantification was performed as Song et al. (2021). The captured images were analyzed for Amylo-Glo intensity in ImageJ Fiji 2.0.0 (https://imagej.net/Fiji). We adjusted the images to 8-bit and subtracted the background. We then selected the proper threshold of signals (maximum: 255, minimum: 0). When all the Amylo-Glo signals in the section has become red, we quantified the Integrated density (total intensity within threshold). The Amylo-Glo intensity was measured in both hemispheres in two to five sections and were averaged for each mouse. All measurements were quantified by a researcher who was blind to the group identity of the sample.

### Experimental design and statistical analysis

For male mice the experimental design was a 2 × 2 between-subjects factorial design, with age as one factor (36 vs 52 weeks old) and genotype as the other (wild- type vs J20). For females all mice were 36 weeks old so comparisons were made only between genotypes. All data were analyzed using orthogonal contrasts controlling the per-contrast error rate at α = 0.05 according to the procedure described by Hays (1973). Lever press acquisition data included “day” as a repeated measure for which both the main effects and interactions with between-subjects factors are reported. If significant interactions were identified then follow-up simple effects were also reported. Test data included “lever” (Valued vs Devalued) as the repeated measure instead of day. Again, both main effects and interactions with between-subjects factors are reported, with follow-up simple effects if significant interactions were detected. For test data, we report both the raw scores and those scores as a percentage of baseline responding. Baseline responding is defined as the lever press rates per minute on each lever, averaged across the 2 d of training immediately before test (i.e., for the 4-d test this was days 3–4, for the 8-d test this was days 7–8).

The data files for these experiments including all details of statistical analyses can be accessed at the following DOI: 10.17 605/OSF.IO/JXYC9.

## Results

### Goal-directed action is initially impaired for J20 male mice at 36-week-old and for all 52-week-old male mice regardless of genotype

Thirty-nine male mice, comprised of four groups: 36-week-old wild-types (*n* = 8), 36-week-old J20 mice (*n* = 9), 52-week-old wild-types (*n* = 8), and 52-week-old J20s (*n* = 14), were used for this study. Ages were taken at the beginning of behavioral training. We chose to test mice at 36 weeks because this was the earliest age at which Aβ plaque load was previously reported to significantly differentiate from wild-types (Wright et al., 2013), and 52 weeks was chosen to determine whether the behavioral profile of these animals changed as animals aged and their neuropathological features progressed.

**Figure 1.**
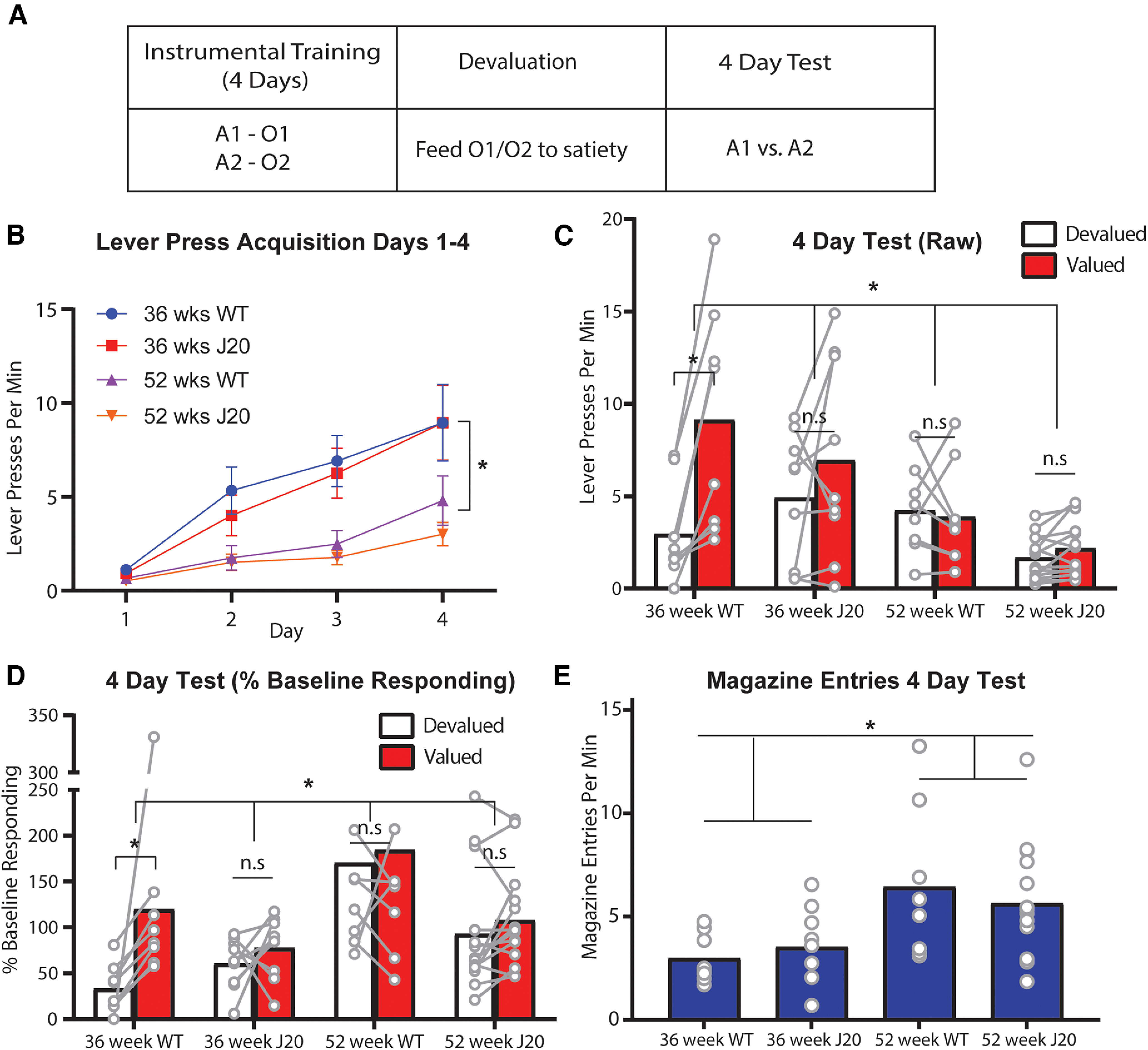
Outcome devaluation performance was initially impaired in 36-week-old male J20 mice and in all 52-week-old mice regardless of genotype. ***A***, Design of outcome devaluation. ***B***, Lever press rates (±SEM) during days 1–4 of lever press acquisition. ***C***, Lever press rates during the 4-d devaluation test. ***D***, Lever press rates during the 4-d devaluation test displayed as a percentage of baseline responding. ***E***, Magazine entries during the 4-d devaluation test. A = action, O = outcome. *p < .05, n.s. = non-significant.

The outcome devaluation procedure used is shown in Figure 1*A*. As mentioned, we based the design on the prior study by Bradfield et al. (2020) that was conducted in rats. However, our pilot studies revealed that alterations were necessary to translate this task to mice because 1−2 d of lever press training was in sufficient for even wild-type mice to demonstrate intact devaluation. Therefore, we initially trained mice to press a left and right lever for pellet and sucrose outcomes (in Fig. 1*A*, the actions are referred to as A1 and A2, respectively, and outcomes are O1 and O2, respectively, counterbalanced) over 4 d on an increasing ratio schedule (see Materials and Methods). Half of the mice in each group received the left lever paired received the opposite arrangement. Mice then received the initial devaluation test, which we hereafter refer to as the “4-d test,” during which one of the two outcomes (O1) was prefed to satiety to reduce its value. Mice were subsequently given a choice between the two levers in extinction (i.e., both levers were extended but presses did not earn any outcomes). The test was repeated on the following day with the alternate outcome, O2. Animals with intact goal-directed action should respond on the lever associated with the still-valued outcome (Valued > Devalued; in Fig. 1*A*, A1 > A.2). We expected devaluation to be intact for wild-type animals of either age, but that it would be impaired (i.e., Valued = Devalued) for J20 mice.

Lever press rates during days 1–4 of acquisition are shown in Figure 1*B*. All mice increased lever pressing over days although this increase was accelerated for the 36-week-old mice relative to the 52-week-old mice. In support of these observations, there was a linear main effect, *F*_(1,35)_ = 72.628, *p* < 0.00001, and a main effect of age, *F*_(1,35)_ = 15.694, *p* = 0.0003, but no main effect of genotype, *F* < 1. Importantly, all linear simple effects were significant (smallest *F*_(1,35)_ = 4.978, *p* = 0.032, for 52-week-old J20s) demonstrating that mice in each group did increase their lever pressing across days, indicating intact lever press acquisition for all groups.

Lever pressing during the 4-d devaluation test is shown in Figure 1*C*. As expected, devaluation was intact (Valued > Devalued) for 36-week-old wild-types, and impaired (Valued = Devalued) for J20 mice of both ages. Unexpectedly, however, devaluation performance was also impaired for 52-week-old wild-type mice. Statistically, there was a significant three-way (genotype × age × devaluation) interaction, *F*_(1,35)_ = 5.059, *p* = 0.031, suggesting that devaluation performance differed between J20 mice and wild-type mice at 36 weeks old, but not at 52 weeks old. This interaction can be explained by the significant two-way genotype × devaluation interaction at 36 weeks, *F*_(1,35)_ = 6.399, *p* = 0.016, but no such interaction at 52 weeks, *F* < 1. Specifically, at 36 weeks old, devaluation was intact (Valued > Devalued) for wild-types, simple effect *F*_(1,35)_ = 26.913, *p* < 0.00001, but not J20s (Valued = Devalued) at this age, simple effect, *F*_(1,35)_ = 3.294, *p* = 0.078, whereas devaluation was impaired and for both genotypes at 52 weeks old, as *F* < 1 for both simple effects.

Across this test, there was also a main effect of age *F*_(1,35)_ = 9.821, *p* = 0.003 (but not of genotype, *F*_(1,35)_ = 1.369, *p* = 0.25) suggesting that the younger mice, regardless of genotype, displayed higher overall levels of lever pressing (i.e., averaged across levers). This finding, along with the main effect of age observed during lever press acquisition, raises the possibility that the older mice simply experienced a general motor impairment, as opposed to a specific impairment in their ability to exert goal-directed control. This conclusion is challenged, however, by the fact that 52-week-old mice made significantly more of the competing motor responses: head entries into the food magazine (Fig. 1*E*), relative to 36-week-old mice averaged over genotype. This is supported by a main effect of age, *F*_(1,35)_ = 10.416, *p* = 0.003, no main effect of genotype, and no genotype × age interaction, both *F*s < 1.

Moreover, when lever press data from the test is calculated as a percentage of baseline responding, as shown in Figure 1*D*, this again produces a main effect of age, *F*_(1,35)_ = 6.488, *p* = 0.015, but this is actually in the opposite direction, with 52-week-old mice responding >36-week-old mice relative to baseline. Yet the distribution of their responding remains equal between the valued and devalued levers. This is supported by a significant 2-way genotype × devaluation interaction for 36-week-old mice, *F*_(1,35)_ = 4.608, *p* = 0.039, whereas there is no such interaction at 52 weeks, *F* < 1, and once again the 36-week-old wild-types are the only group for whom a significant simple effect (Valued > Devalued) was observed, *F*_(1,35)_ = 13.508, *p* = 0.001, whereas all other simple effects, *F* < 1. Together with magazine entry data, this result suggests that it is goal-directed control specifically, rather than motor control more generally, that is impaired in 52-week-old mice.

### Goal-directed action is intact for all male mice after additional lever press training, regardless of age or genotype

As mentioned, we expected the devaluation impairment in J20 mice to be overcome with additional lever press training. We tested this by giving mice an additional 4 d of lever press training (i.e., 8-d lever press training in total), and then administering a second devaluation test which we hereafter referred to as the “8-d test.” On this test we expected devaluation performance to be intact for all mice, regardless of age or genotype.

**Figure 2.**
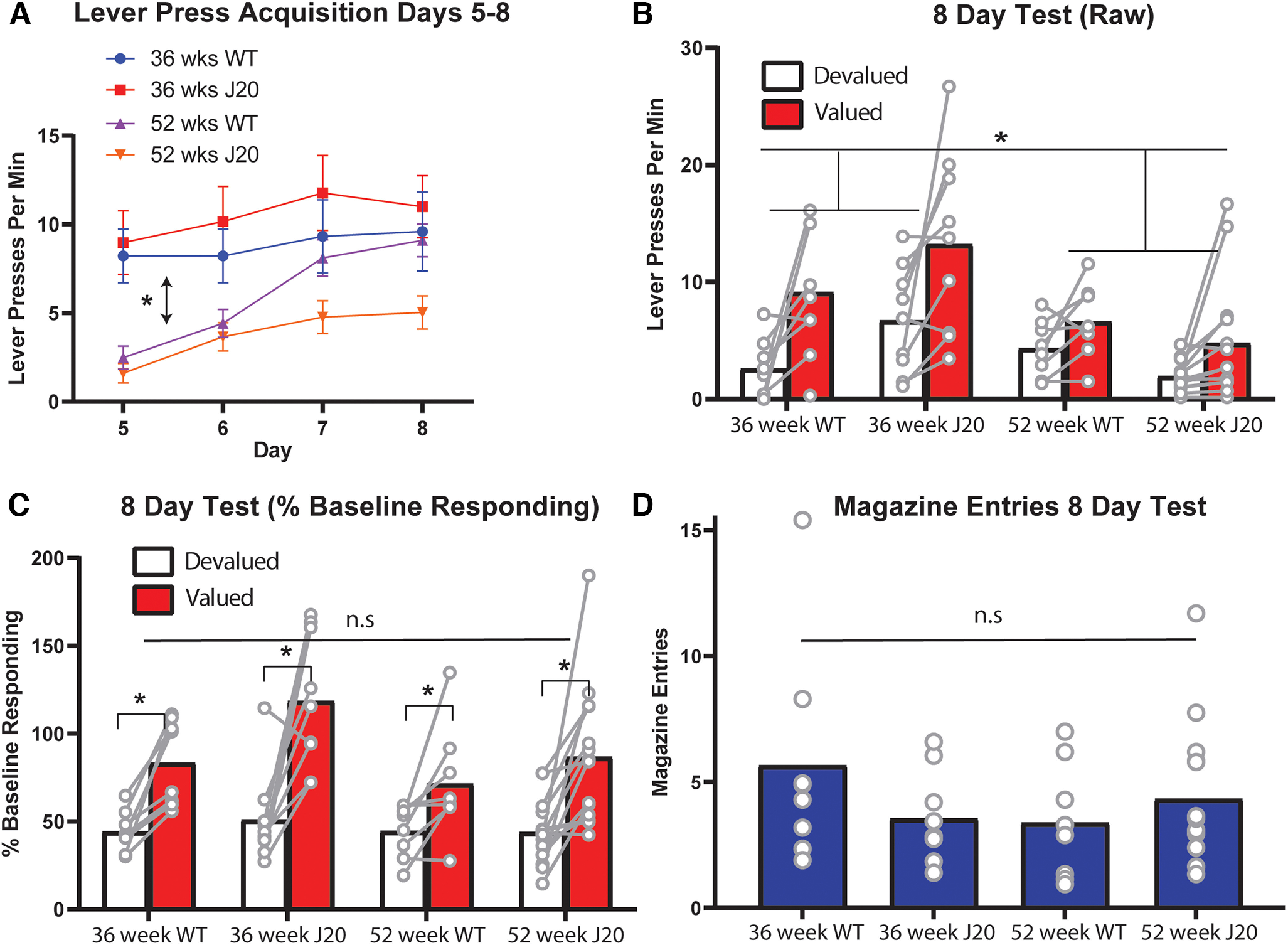
Outcome devaluation was intact in the 8-d devaluation test for all male mice, regardless of age or genotype. ***A***, Lever press rates (±SEM) during days 5–8 of lever press acquisition. ***B***, Lever press rates during the 8-d devaluation test. ***C***, Lever press rates during the 8-d devaluation test displayed as a percentage of baseline responding. ***D***, Magazine entries during the 8-d test. *p < .05, n.s. = non-significant.

Lever press rates during the additional lever press training (days 5–8) is shown in Figure 2*A*. Once again, 36-week-old mice lever pressed >52 week olds, as supported by a main effect of age, *F*_(1,35)_ = 14.049, *p* = 0.001, but lever pressing did not differ according to genotype, main effect, *F* < 1. Only 52-week-old mice increased lever press rates across days 5–8, as supported by a linear main effect, *F*_(1,35)_ = 39.196, *p* = 0.00, that interacted with age, *F*_(1,35)_ = 8.333, *p* = 0.007. Follow-up simple effects reveal that this interaction comprises linear increases in both 52-week-old groups: wild-types, *F*_(1,35)_ = 32.913, *p* < 0.00001 and J20s, *F*_(1,35)_ = 13.436, *p* = 0.001, but no such increase for either group at 36 weeks of age: wild-types, *F*_(1,35)_ = 1.622, *p* = 0.211 and J20s, *F*_(1,35)_ = 3.912, *p* = 0.056 (although the latter effect could be considered marginal).

Lever pressing during the 8-d test is shown in Figure 2*B*. From this figure, devaluation performance appears intact (i.e., Valued > Devalued) for all groups despite the persistence of lower overall press rates in the 52 week olds. These observations are supported by a main effect of age, *F*_(1,35)_ = 9.152, *p* = 0.005, but not of genotype, *F* < 1, and a main effect of devaluation, *F*_(1,35)_ = 27.95, *p* < 0.00001. This time, however, there was no three-way interaction, *F* < 1, and no two-way interactions at either 36 weeks or 52 weeks, both *F*s < 1. There was, however, a two-way age × devaluation interaction, *F*_(1,35)_ = 5.527, *p* = 0.024, suggesting that performance was superior for 36 week olds relative to 52 week olds.

Nevertheless, this result appears to be a consequence of the lower lever press rates in the older mice rather than a difference in the ability to express goal-directed action control, because once test performance was expressed as a percentage of baseline responding these differences disappeared. These data are shown in Figure 2*C*. Specifically, there was a main effect of devaluation, *F*_(1,35)_ = 49.607, p < . 00,001, but this time there was no age × devaluation interaction, *F*_(1,35)_ = 2.177, *p* = 0.149, suggesting that devaluation was indeed intact for all mice. Moreover, and in contrast to the 4-d test, there were no group differences in magazine entries on this test as shown in Figure 2*D*, all *F*s < 1.

Taken together with the results of the day 4 test, these findings suggest that goal-directed action is initially impaired in J20 mice at 36 weeks old, and for mice of both genotypes at 52 weeks old, but that it is intact for all mice after additional lever press training.

### Outcome selective reinstatement is impaired for 52-week-old J20 males relative to wild-types

**Figure 3.**
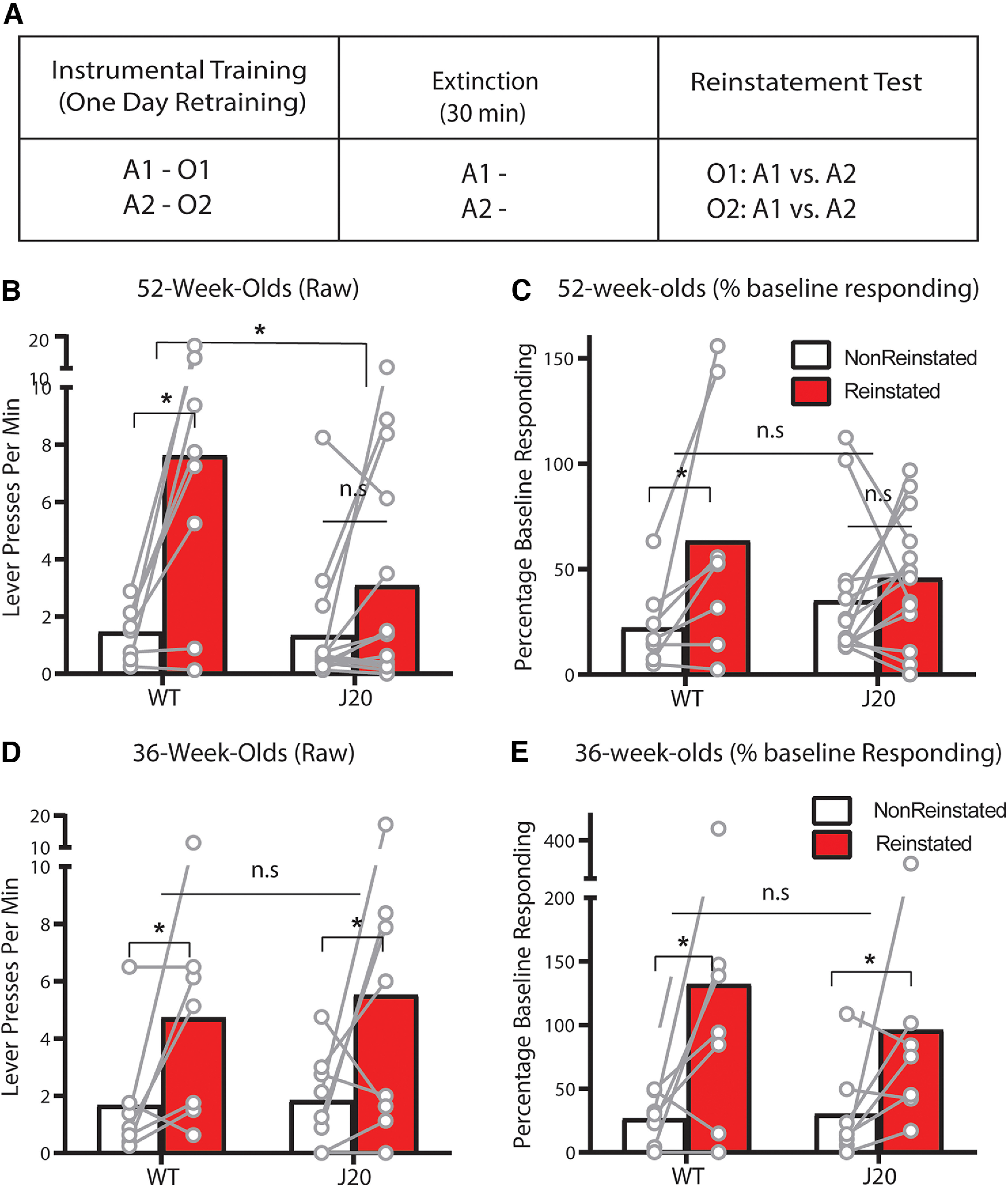
Outcome-selective reinstatement was impaired in 52-week-old J20 male mice but was unaltered in J20 male mice at 36 weeks old. ***A***, Design of the outcome selective reinstatement procedure. ***B***, Lever press rates during outcome selective reinstatement testing of 52 week olds. ***C***, Lever press rates during the same test as a percentage of baseline responding. ***D***, Lever press rates during outcome selective reinstatement testing of a separate cohort of 36 week olds. ***E***, Lever press rates during the same test as a percentage of baseline responding. A = action, O = outcome. *p < .05, n.s. = non-significant.

So far, we have detected a wild-type/J20 difference in initial goal-directed control for 36-week-old mice but have failed to detect any genotypic differences for 52 week olds. We therefore added another test of choice behavior, outcome-selective reinstatement, to determine whether this lack of difference was general across tasks. Following the 8-d devaluation test, 52-week-old mice were retrained on lever pressing for 1 d then subject to reinstatement testing. The design is shown in Figure 3*A*. On test, animals were first exposed to 30 min of extinction on both levers, after which mice received two unsignaled presentations of each outcome in the following order: sucrose, pellets, pellets, sucrose, each separated by a further 4 min of extinction. If outcome-selective reinstatement were intact, animals should selectively reinstate responding on the lever that had earned that outcome during training. For instance, sucrose presentations should elicit pressing on the sucrose lever, and vice versa.

Test data are shown in Figure 3*B*. This time, we did observe a genotypic difference in 52 week olds because selective reinstatement was intact for wild-types (Reinstated > Nonreinstated) but impaired for J20s (Reinstated = Nonreinstated). Statistically, there was a main effect of reinstatement, *F*_(1,20)_ 18.716, *p* = 0.00012, no main effect of genotype, *F*_(1,20)_ = 3.379, *p* = 0.081, and a genotype × reinstatement interaction, *F*_(1,20)_ = 5.526, *p* = 0.029, supported by a significant simple effect for wild-types, *F*_(1,20)_ = 17.514, *p* = 0.00018, but not J20s, *F*_(1,20)_ = 2.683, *p* = 0.117. As we did for devaluation, we again examined the same data as a percentage of baseline responding (Fig. 3*C*) and there was again a main effect of reinstatement, *F*_(1,20)_ = 7.416, *p* = 0.013, however the genotype × reinstatement interaction was no longer significant, *F*_(1,20)_ = 2.589, *p* = 0.123. Nevertheless, the percent baseline transformation was not sufficient to rescue the reinstatement effect for the J20s, as their simple effect *F* < 1, whereas the simple effect for wild-types was still significant, *F*_(1,20)_ = 7.373, *p* = 0.013.

Unfortunately, at the time the decision was made to test selective reinstatement, several of the 36-week-old male mice had already been culled. We thus decided to test reinstatement in an independent cohort of 36-week-old males (wild-types: *n* = 7, J20s: *n* = 8), and confirmed that outcome selective reinstatement was intact for both wild-types and J20s at this age, as shown in Figure 3*D*. Statistically, there was a main effect of reinstatement, *F*_(1,13)_ = 6.626, *p* = 0.023, no main effect of genotype, and no genotype × reinstatement interaction, both *F*s < 1. The same data are shown as a percentage of baseline responding in Figure 3*E*. As with the raw data, there was a main effect of reinstatement, *F*_(1,12)_ = 5.955, *p* = 0.031 that did not interact with genotype, *F* < 1.

This result demonstrates that there is one aspect of choice behavior, selective reinstatement, that is impaired in 52-week-old J20s relative to wild-types. Together with the devaluation, this result suggests that after 8 d of training 52-week-old J20 mice are capable of using response-outcome associations, but not outcome-response associations to inform their action selection (Balleine and Dickinson, 1998; Ostlund and Balleine, 2007; Abiero et al., 2022).

### Goal-directed action is initially impaired for 36-week-old J20 female mice but is intact after additional lever press training

Nineteen females, *n* = 9 wild-types and *n* = 10 J20s, were trained on a modified version of the outcome devaluation procedure previously described. Specifically, all procedures were identical except that the advancement through the ratio requirements for lever pressing was slower, and outcome devaluation tests were conducted for 5 min each rather than 10 min to reduce extinction due to the lower levels of lever pressing in females relative to males (see Materials and Methods for details).

**Figure 4.**
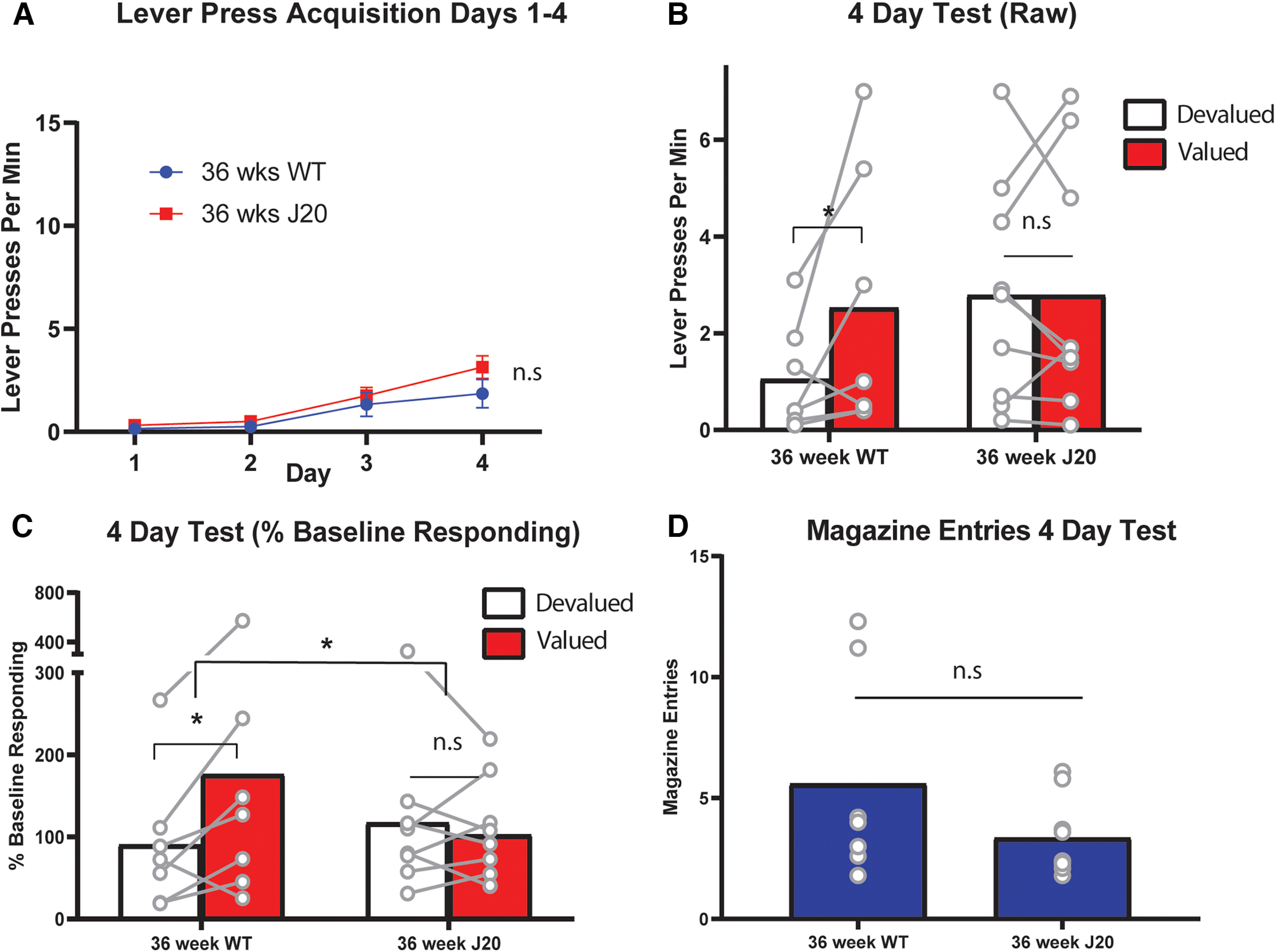
Outcome devaluation performance is initially impaired in female J20 mice at 36 weeks old. ***A***, Lever press rates (±SEM) during days 1–4 of acquisition. ***B***, Lever press rates during the 4-d devaluation test. ***C***, Lever press rates during the 4-d devaluation test displayed as a percentage of baseline responding. ***D***, Magazine entries during the 4-d test. *p < .05, n.s. = non-significant.

Lever press acquisition for females is shown in Figure 4*A*. Three mice were excluded from the analysis (two WT and one J20) for failing to perform more than a single lever press during initial acquisition. Final sample size was therefore *n* = 7 wild-types and *n* = 9 J20s. From Figure 4*A*, lever pressing increased similarly for both groups across days as confirmed by a linear main effect, *F*_(1,14)_ = 30.123, *p* < 0.00001, no main effect of group, *F*_(1,14)_ = 1.663, *p* = 0.218, and no group × linear interaction, *F*_(1,14)_ = 1.511, *p* = 0.239.

Performance on the 4-d devaluation test is shown in Figure 4*B*. This figure reveals a similar pattern of responding to that observed in the 36-week-old males: intact devaluation in wild type mice (Valued > Devalued) and impaired devaluation in J20s (Valued = Devalued). Statistically, however, this result was not as robust as that observed for male mice (possibly because of lower sample size) as there was no main effect of group, *F* < 1, no main effect of devaluation, *F*_(1,14)_ = 2.868, *p* = 0.112, and no group × devaluation interaction, *F*_(1,14)_ = 2.868, *p* = 0.112. Nevertheless, once test data were transformed to a percentage of baseline responding, as shown in Figure 4*C*, although there was still no main effect of group, *F* < 1, or of devaluation, *F*_(1,14)_ = 3.216, *p* = 0.095, there was a significant group × devaluation interaction, *F*_(1,14)_ = 4.668, p .049. Follow-up analysis revealed a significant simple effect in the wild types, *F*_(1,14)_ = 6.948, *p* = 0.02, suggesting that they pressed the valued lever more than the devalued lever, but no such simple effect for the J20s, *F* < 1, demonstrating that they pressed both levers equally. As shown in Figure 4*E*, magazine entries did not differ between groups on this test, *F*_(1,14)_ = 2.084, *p* = 0.171.

**Figure 5.**
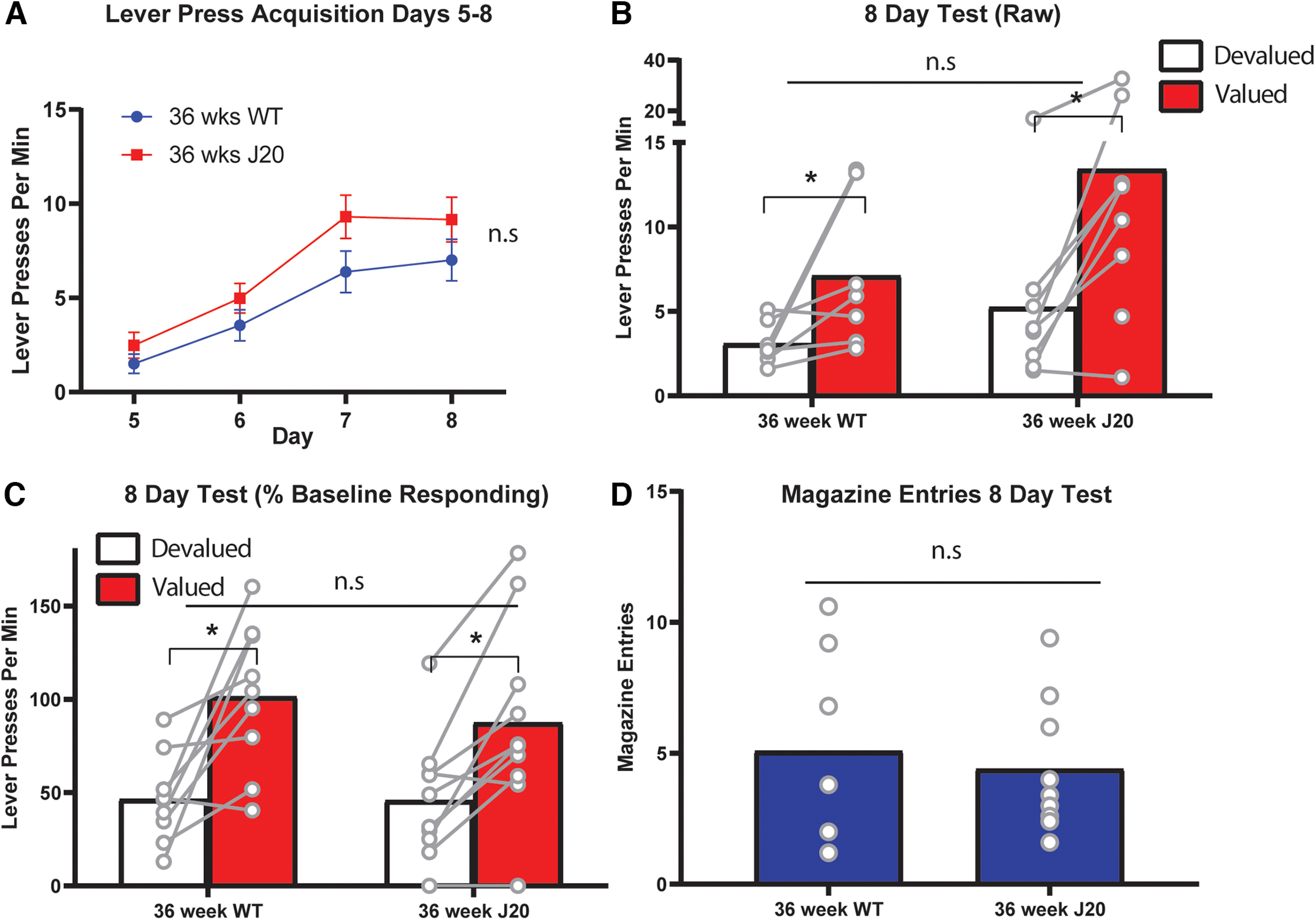
Outcome devaluation performance was intact for all female mice, regardless of genotype, after 8 d of lever press training. ***A***, Lever press rates (+/–) SEM during days 5–8 of acquisition. ***B***, Lever press rates during the 8-d devaluation test. ***C***, Lever press rates during the 8-d devaluation test as a percentage of baseline responding. ***D***, Number of entries into the food receptacle (magazine) averaged over days 5–8. *p < .05, n.s. = non-significant.

Female mice were next subject to four more days of lever press training (i.e., 8 d total) and retested for devaluation performance to determine if, like male J20s, the female J20 mice could also overcome the initial deficit in goal-directed action with additional training. Mice in both groups continued to acquire lever pressing across days 5–8 as shown in Figure 5*A* and supported by a linear main effect, *F*_(1,14)_ = 108.45, *p* < 0.00001. This increase was equivalent because there was no main effect of group, *F*_(1,14)_ = 2.271, *p* = 0.154, and no group × linear interaction, *F*_(1,14)_ = 1.408, *p* = 0.255.

Data from the 8-d test is shown in Figure 5*B*. It is clear from this figure that devaluation was intact (Valued > Devalued) for both groups, and this is supported by a main effect of devaluation, *F*_(1,14)_ = 15.862, *p* = 0.001, no main effect of group, *F*_(1,14)_ = 2.335, *p* = 0.149, and no group × devaluation interaction *F*_(1,14)_ = 1.843, *p* = 0.196. Although not statistically supported, it does appear from Figure 5*B* as though mice in the J20 group were responding more overall than wild types on this test. However, if we again examine test performance as a percentage of baseline responding, as shown in Figure 5*C*, this difference dissipates. Specifically, there is a main effect of devaluation, *F*_(1,14)_ = 30.582, *p* < 0.00001, no main effect of group, *F* < 1, and no group × devaluation interaction *F*_(1,14)_ = 1.129, *p* = 0.306. Magazine entries did not differ between groups on this test, *F* < 1, as shown in Figure 5*D*.

Together with data from the 4-d test, these results suggest that goal-directed action is initially impaired in female J20s relative to wild types but that this impairment can be overcome with additional lever press training, in a similar manner to the results for male mice of 36 weeks old. This suggests that the deficit is general to both sexes.

### Outcome-selective reinstatement is intact for all 36-week-old female mice

**Figure 6.**
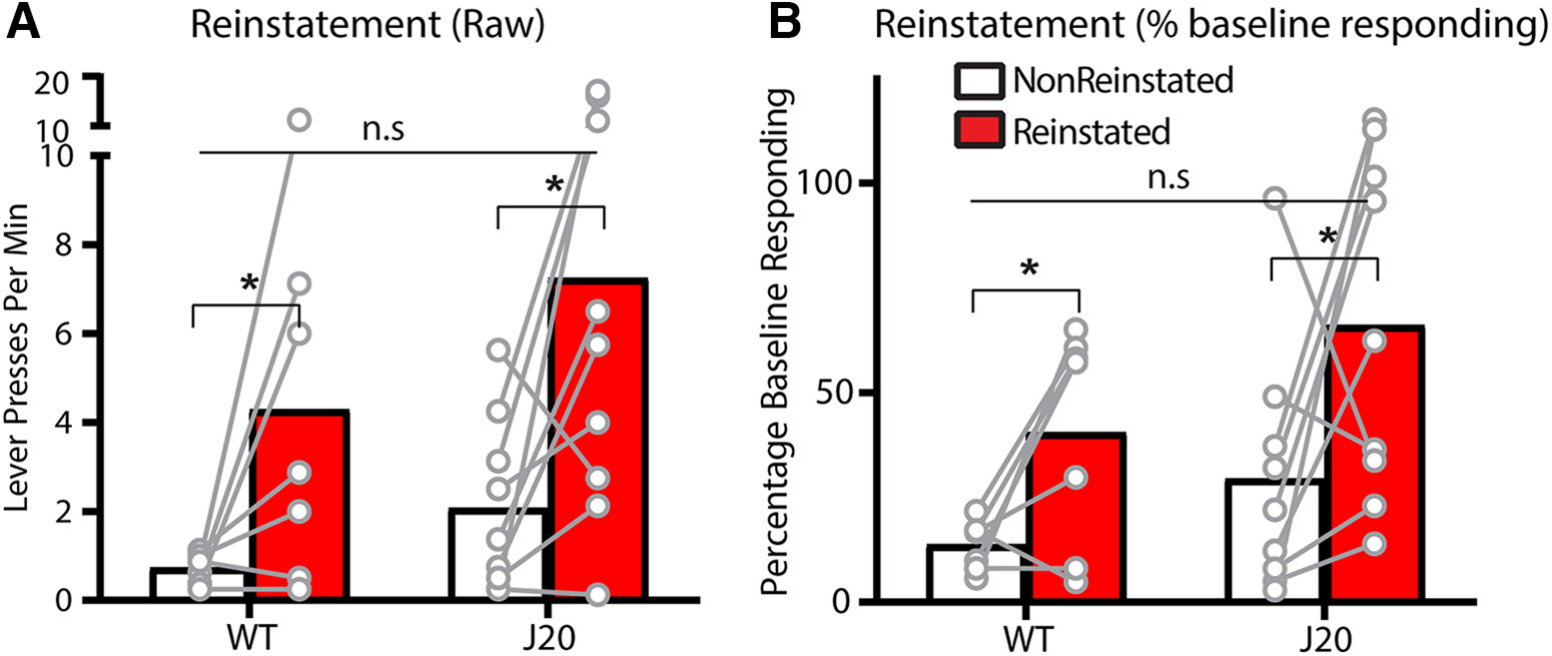
Outcome-selective reinstatement was intact for all females, regardless of genotype. ***A***, Lever press rates for female 36-week-old mice during outcome selective reinstatement testing. ***B***, Lever press rates on the same test expressed as a percentage of baseline responding. *p < .05, n.s. = non-significant.

We also tested female mice for their performance on outcome-selective reinstatement, data from this test is shown in Figure 6*A*. Outcome-selective reinstatement (Reinstated > Nonreinstated) was intact for all females, regardless of genotype. Specifically, there was a main effect of reinstatement, *F*_(1,14)_ = 10.49, *p* = 0.006, but no main effect of group, *F*_(1,14)_ = 2.278, *p* = 0.153 and no group × reinstatement interaction, *F* < 1. The same data are presented as a percentage of baseline responding in Figure 6*B*. Once again, there was a main effect of reinstatement, *F*_(1,14)_ = 8.062, *p* = 0.013 which did not interact with genotype, *F* < 1.

### Dorsal CA1 microglial (IBA1) expression increased with age but not genotype, and negatively correlated with initial devaluation performance

**Figure 7.**
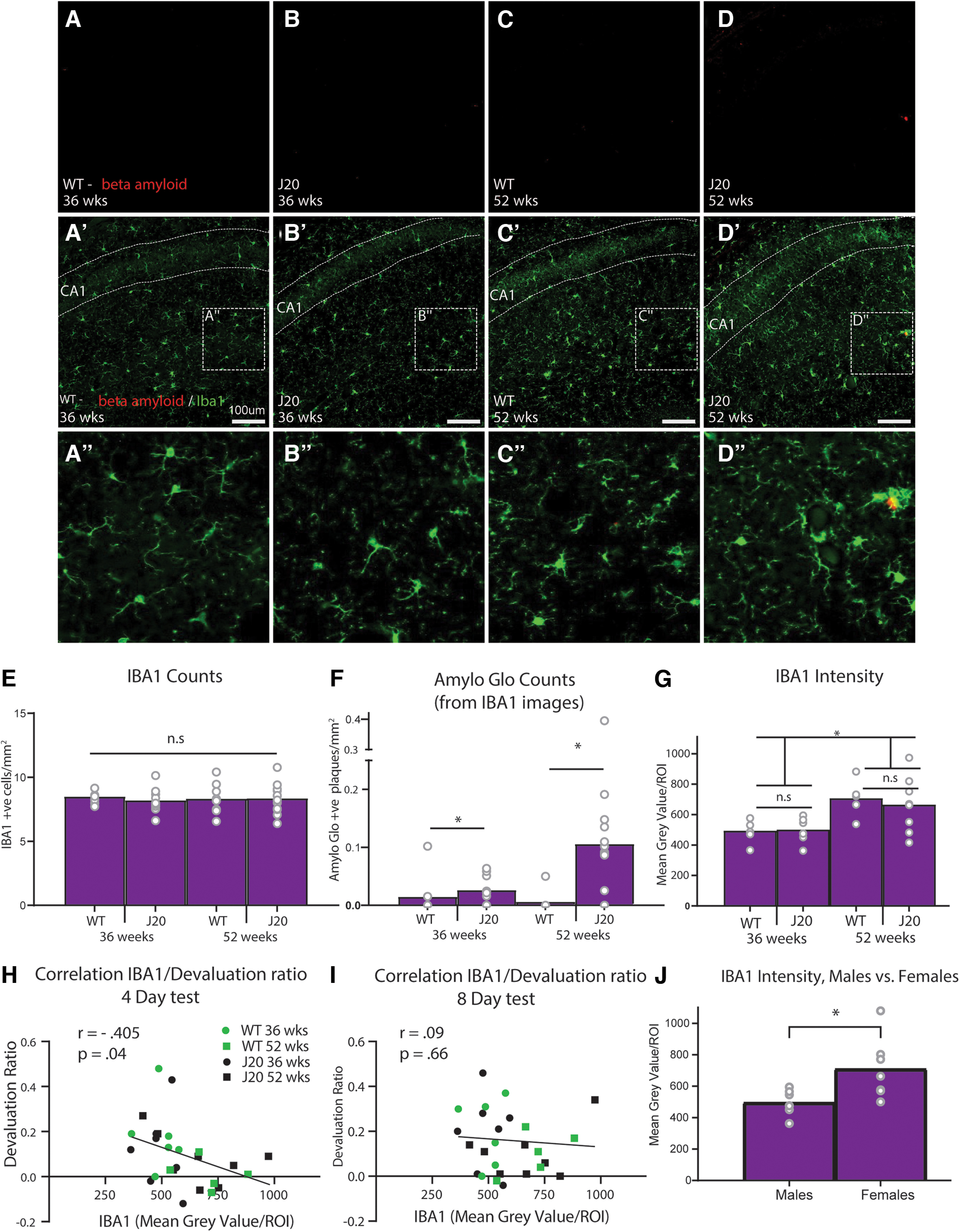
Quantification of IBA1 and Amylo Glo expression in the dorsal CA1 of male mice. ***A–D***, Representative photomicrographs of male dorsal CA1 stained with Amylo Glo (red) in each group. Scale bar: 100 μm. ***A’–D’***, Representative photomicrographs of male dorsal CA1 stained with Amylo Glo (red) and IBA1 (green) in each group. ***A’’–D’’***, Enlarged inset. ***E***, Number of cells positively immunostained for IBA1 per mm^2^. ***F***, Number of plaques positively immunostained for Amylo Glo (taken from IBA1 images) per mm^2^. ***G***, IBA1 intensity quantification. ***H***, Correlation between IBA1 intensity and devaluation ratio from the 4-d test. ***I***, Correlation between IBA1 intensity and devaluation ratio from the 8-d test. ***J***, IBA1 intensity quantification for 36-week-old J20 males versus 36-week-old J20 females. *p < .05, n.s. = non-significant.

Because we detected robust and consistent behavioral deficits in J20 animals, we decided to follow-up with immunohistochemical analyses of amyloid plaque markers and markers of putative neuroinflammation to detect the potential mechanisms that might underlie these deficits. Following behavioral testing, dorsal hippocampal sections were immunostained with IBA1, a protein that is only present in microglia and is upregulated when those cells are activated (Ito et al., 1998) as occurs during an inflammatory response (Harry, 2013). Sections were co-stained with Amylo-Glo, to identify amyloid plaques (Schmued et al., 2012), see Materials and Methods for details. Representative examples of these stains from sections taken from wild type and J20 mice at 36 and 52 weeks of age, respectively, are shown in Figure 7*A–D*.

First, we analyzed sections from male mice. Initially we quantified IBA1 and Amylo Glo expression by conducting automated counts using ImageJ software (see methods for details), but failed to find any group differences in IBA1 positive cells, *F*s < 1, shown in Figure 7*E*. As expected, however, plaques stained for Amylo Glo were significantly higher in J20s than wild-types (Fig. 7*F*), as evidence by a main effect of genotype, *F* = 6.318, *p* = 0.017, but not of age, *F* = 2.608, *p* = 0.116. Despite some co-localization between microglia and plaques, as can be seen in Figures 7*D*, the expression of IBA1 and Amylo-Glo for J20 mice did not significantly correlate, *r* = 0.047, *p* = 0.835. It should be noted that the “plaques” that were identified for two WT mice (Fig. 7*F*) were likely background as they did not co-localize with microglia as would be expected.

Following the failure to detect any group differences in microglia using cell counts, additional brain sections for each mouse were taken and immunostained for IBA1 and then imaged using a different, intensity-based quantification for which mean gray values were obtained for each marker. Our reasoning was that although genotype and/or age may not lead to changes in number of cells, they could alter cell morphology in a way that might be detected by an intensity-based but not count-based methodology. The results of this quantification are shown in Figure 7*G*. When analyzed according to intensity, IBA1 expression was found to differ according to age but still did not differ according to genotype, as confirmed by a main effect of age (52 weeks > 36 weeks), *F*_(1,22)_ = 14.192, *p* = 0.001, but not genotype, *F* < 1, and no age × genotype interaction, *F* < 1.

We next correlated IBA1 expression with devaluation performance on both the 4-d and the 8-d tests. To calculate this correlation, we used a “devaluation score” to ensure that any correlation detected was not driven by baseline differences in lever press responding per se. For this score, we first calculated suppression ratio (SR) scores on each of the levers (i.e., valued and devalued) according to the following equation:

$$SR=\frac{Lever\;press\;rate\;on\;test}{\left(Lever\;press\;rate\;on\;test\;+\;Lever\;press\;rate\;during\;training\right)}.$$

We then subtracted the Devalued SR from the Valued SR for each test separately, such that a positive devaluation score indicated more responding on the valued than the devalued lever, indicative of intact goal-directed action, and a zero or negative score indicated impaired goal-directed action. This score was correlated with microglial expression.

As shown in Figure 7*H*,*I*, we found that IBA1 expression (mean gray value) negatively correlated with devaluation ratio scores taken from the 4-d test, *r* = −0.405, *p* = 0.04 (Fig. 7*H*) but not the 8-d test, *r* = 0.09, *p* = 0.66 (Fig. 7*I*). This suggests that increased microglial expression in the dorsal CA1 is associated with poorer devaluation performance on the initial test but not after extended training, which could imply that initial deficits in goal-directed action are associated with neuroinflammation in this region. Amylo-Glo counts did not correlate with any behavioral measure on either test, smallest *p* = 0.157.

For female mice, we were only able to image and quantify sections from three to four wild-types which meant that the sample size was not sufficiently large to allow an accurate statistical analysis of group differences. Further, there were no significant correlations with these markers and any behavioral measures for female mice, although this was again likely because of low sample size. The quantification and correlation values for these mice are included in the data files at DOI: 10.17 605/OSF.IO/JXYC9, if researchers are interested in observing the numerical differences and/or conducting their own analyses. Nevertheless, because immunohistochemistry and subsequent quantification for the intensity-based analyses were conducted on all sections from male and female mice at the same time using the same techniques and parameters, we were able to directly compare levels of IBA1 between 36-week-old J20 animals of each sex (*n* = 7 females, *n* = 7 males) as shown in Figure 7*J*. IBA1 expression was significantly higher in female J20s relative to male J20 mice at this age, *F*_(1,12)_ = 7.096, *p* = 0.021.

### Dorsal CA1 astrocytic (GFAP) expression in male mice increased with age but not genotype but did not correlate with any behavioral measure

**Figure 8.**
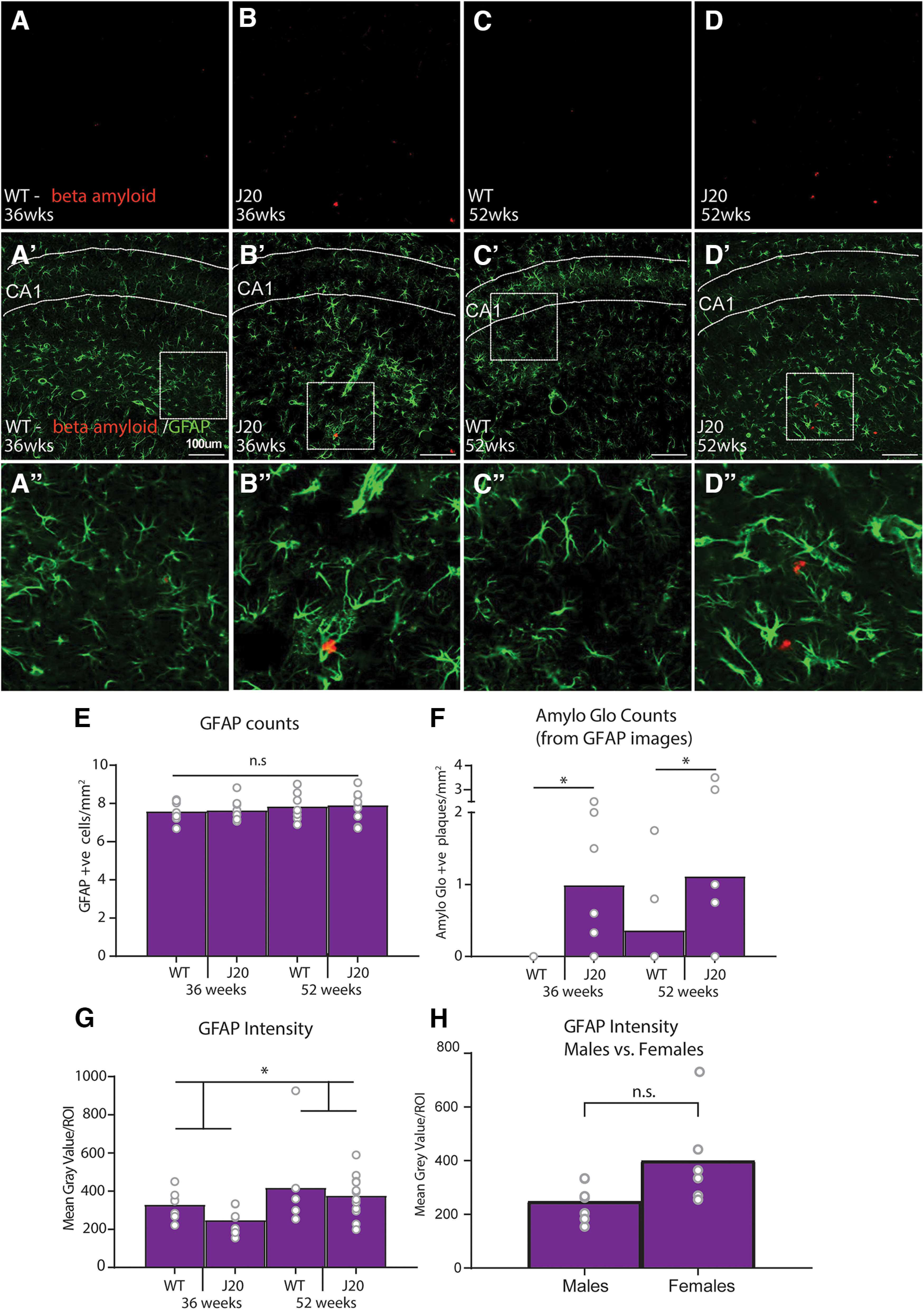
Quantification of GFAP and Amylo-Glo expression in the dorsal CA1 of male mice. ***A–D***, Representative photomicrographs of male dorsal CA1 stained with Amylo Glo (red) in each group. Scale bar: 100 μm. ***A’–D’***, Representative photomicrographs of male dorsal CA1 stained with Amylo Glo (red) and GFAP (green) in each group. ***A’’–D’’***, Enlarged inset. ***E***, Number of cells positively immunostained for GFAP per mm^2^. ***F***, Number of plaques positively immunostained for Amylo-GLo (taken from GFAP images) per mm^2^. ***G***, GFAP intensity quantification. ***H***, GFAP intensity quantification, 36-week-old J20 males versus 36-week-old J20 females. *p < .05, n.s. = non-significant.

Separate sections were taken from the same animals and co-stained with GFAP, a common marker for astrocytes, as well as with Amylo-Glo, to identify amyloid plaques (see Materials and Methods). Representative examples of these stains from wild-type and J20 mice at both 36 and 52 weeks of age, respectively, are shown in Figure 8*A–D’’*. Sections were imaged and quantified as previously described. Analyses of cell counts for male mice are shown in Figure 8*E*,*F*. Again, counts of GFAP positive cells (Fig. 8*E*) did not differ according to genotype, *F* < 1, or age, *F*_(1,30)_ = 1.268, *p* = 0.269, whereas Amylo Glo positive plaques (Fig. 8*F*) were significantly higher in J20s relative to wild-types, *F*_(1,30)_ = 8.058, *p* = 0.008, but not age, *F* < 1. Again, overall numbers of cells/plaques positive for GFAP and Amylo-Glo did not significantly correlate with each other, *r* = 0.12, *p* = 0.61. In addition, although “plaques” were identified in two WT mice, these were also likely background staining.

We next conducted a separate immunostain and intensity-based analysis for GFAP with the hope of detecting morphology differences not obtained using cell counts. The results are shown in Figure 8*G*. Like IBA1, GFAP expression did not differ according to genotype but was higher in the older, 52-week-old mice relative to 36 week olds. Indeed, there was no main effect of genotype *F*_(1,28)_ = 1.506, *p* = 0.23, but there was an effect of age, *F*_(1,28)_ = 4.549, *p* = 0.042, that did not interact with genotype *F* < 1. GFAP expression did not significantly correlate with any behavioral measure on either test, all *p*s > 0.05 (see data files for more detail).

Although low sample size in wild-type females again precluded an analysis of group differences of GFAP in the female cohort, the quantification and correlation values for these mice are again included in the data files at DOI: 10.17 605/OSF.IO/JXYC9. However, like with IBA1 analysis, because all procedures for the intensity-based analyses were conducted on all sections from male and female mice at the same time for 36-week-old J20 animals of each sex (*n* = 7 males, *n* = 6 females), we were able to compare these directly as shown in Figure 8*H*. As with IBA1, GFAP expression was also increased in female J20s relative to male J20 mice at this age, although statistically the effect was only marginal, *F*_(1,11)_ = 4.39, *p* = 0.06.

## Discussion

Here, we report several experiments demonstrating that hAPP-J20 mice of both sexes exhibit an initial impairment in goal-directed action that can be overcome with additional training, as do aging male mice. Specifically, for both male and female 36-week-old mice, outcome devaluation was initially impaired for J20s (Valued = Devalued) relative to wild-types (Valued > Devalued), whereas it was impaired for all 52-week-old male mice regardless of genotype. Following 4 d of additional lever press training, outcome devaluation was intact for all mice regardless of age or genotype. Interestingly, from the graphs it appears that several of the individual mice from the “impaired” groups did demonstrate intact devaluation (see Figs. 1*C*, 3*B*), suggesting that some of these mice were able to exhibit goal-directed action. We interpret this as reflective of a variety of cognitive abilities in these mice despite their genotype and/or age, and note that such variability has been observed in older humans, as well as humans with Alzheimer’s disease (Hedden et al., 2013). On a separate test of choice behavior: outcome-selective reinstatement, performance was intact for 36-week-old mice of both sexes and genotypes but was impaired for 52-week-old male J20s relative to age-matched wild-types. Follow-up immunohistochemical analyses of dorsal CA1 hippocampal tissue revealed increased microglial expression (intensity) in 52-week-old males relative to 36-week-old males, regardless of genotype, and this expression negatively correlated with devaluation performance on the 4-d, but not the 8-d, test. Finally, our immunohistochemical markers of microglia (IBA1) and astrocytes (GFAP) were increased in 36-week-old female J20s relative to male J20s of the same age, a result that perhaps mirrors the enhanced prevalence of Alzheimer’s disease in female patients relative to male patients for whom it is approximately twice as frequent (Podcasy and Epperson, 2016; Laws et al., 2018). Together, these results suggest that goal-directed action and choice behavior is altered in an hAPP-J20 preclinical model of Alzheimer’s disease, and that the impairments are associated with dorsal hippocampal neuroinflammation.

It is first worth addressing the puzzling result that our putative neuroinflammatory markers (IBA1 and GFAP) did not differ according to genotype. We had expected to see J20 > wild-type differences in these markers, as has been previously observed (Flores et al., 2018, 2022). There are several things to note here. First, our quantification techniques could be seen as less thorough than the stereological techniques used by some prior researchers (Wright et al., 2013), which could pick up smaller or more nuanced differences in the data. Second, IBA1 is a marker of all microglia, and it may be that only microglia that become activated as part of a (presumed) neuroinflammatory response differ according to the J20 genotype (Wright et al., 2013; Hong et al., 2016). Third, GFAP is not an absolute marker of all nonreactive astrocytes, and although its immunoreactivity is quite strong in the hippocampus as compared with other markers, it does not capture the entire astrocyte population or the complete morphology of the cell (Zhang et al., 2019; Jurga et al., 2021). Finally, other studies report findings that are consistent with current results, having failed to find any such differences in the dorsal CA1 region specifically (Wright et al., 2013; Dekens et al., 2018) or between J20s and age-matched wild-types at 52 weeks old (Ameen-Ali et al., 2019), suggesting that any genotypic differences observed with these markers could be highly region and age specific.

Nevertheless, the behavioral finding that goal-directed action was initially impaired in 36-week-old mice of both sexes parallels previous findings that inactivating the dorsal CA1 region of the hippocampus also initially impairs goal-directed action, but that such actions become hippocampally-independent with additional training or over time (Bradfield et al., 2020). The fact that both J20 and aging male mice also overcame this deficit after additional lever press training suggests that these animals were slower to learn the underlying action-outcome contingencies. It is important to note, however, that this interpretation is somewhat limited by the fact that these manipulations (i.e., genotype and age) were present throughout all phases of the experiment, including acquisition and test phases, making it difficult to confidently separate whether this was an impairment in the initial learning or the expression of goal-directed action. Regardless, the specific pattern of results does suggest that J20 and older male mice did not suffer from some nonspecific reward processing deficit, as such a deficit should have applied equally to performance on the 8-d test when goal-directed action was intact for all animals. Moreover, differences in locomotor activity also cannot account for these results because for groups that pressed the lever at lower levels, we observed a concurrent increase in their competing magazine entries, suggesting that overall locomotor activity was preserved across mice. Instead, our results indicate a specific deficit in the initial acquisition (or expression) of goal-directed action.

To our knowledge, the dorsal CA1 is the only brain region on which goal-directed action is initially dependent, but becomes independent of after multiple days of training. By contrast, lesions and/or inactivations of alternate brain regions, such as posterior dorsomedial striatum (Yin et al., 2005), nucleus accumbens core (Corbit et al., 2001), prelimbic cortex (Corbit and Balleine, 2003), basolateral amygdala (Ostlund and Balleine, 2008), medial orbitofrontal cortex (Bradfield et al., 2015, 2018), and mediodorsal thalamus (Corbit et al., 2003), have all produced sustained impairments in goal-directed action after multiple days of lever press training (although note that devaluation is sometimes intact if the manipulation occurs post-training). This fact, coupled with the negative correlation between dorsal CA1 microglial expression and initial devaluation performance strongly suggests that the goal-directed deficits observed here were also a result of damage to this region. It is possible that future studies will find neuroinflammation in the other brain regions mentioned to be associated with devaluation performance on tests after eight or more days of lever press training, in line with the more sustained roles these regions play in goal-directed action.

We also reported the surprising result that goal-directed action is initially impaired as a result of aging, at least in male mice. That is, devaluation performance was initially impaired for both wild-type and J20 mice at 52 weeks old. Correspondingly, microglial (and to a lesser extent, astrocytic) expression also appeared to increase with age, being higher in 52-week-old male mice relative to their 36-week-old counterparts. Although both behavioral impairments and microglial expression have been previously shown to increase with age (Schilling and Eder, 2015; Matamales et al., 2016), what stands out about current results is the relatively young age at which this occurred. That is, almost all prior studies have identified such differences when comparing younger mice to mice aged 18 months or older, an age that is at least six months older than our oldest mice. If replicable and translatable, this finding could suggest that aging-related changes to brain and behavior might occur earlier than previously thought.

Another interesting aspect of current findings was the impairment in outcome-selective reinstatement that was specific to 52-week-old J20s. This result is interesting for two reasons. First, it highlights the changing nature of the cognitive-behavioral deficits of J20 mice as they age, suggesting that functions that are intact for younger J20 mice later become impaired, mirroring the cognitive decline that occurs in humans as Alzheimer’s progresses. Second, it suggests that the neuroanatomical location of the impairment might also have spread throughout the brain because recent evidence (Abiero et al., 2022) has suggested that outcome-selective reinstatement performance is unlikely to rely on dorsal hippocampus. Despite this, it is not immediately clear what brain region could be mediating this particular effect because, to our knowledge, no brain region has been identified to mediate selective reinstatement but not outcome devaluation (which was intact in these animals on the 8-d test). Thus, this question will remain to be answered by future studies. Regardless, this finding does suggest that selective reinstatement could prove to be a particularly important test for detecting differences between older wild-type mice and transgenic mice engineered to mimic aspects of Alzheimer’s disease.

From a cognitive perspective, it has previously been suggested that the dorsal hippocampus’ role in goal-directed action is reflective of this region’s role in episodic memory (Bradfield et al., 2020; Abiero and Bradfield, 2021). Specifically, it was proposed that although goal-directed action initially relies on episodic memory, it becomes dependent on extra-hippocampally encoded semantic memory over time. Such a theory fits neatly with current results because episodic memory impairments are well-known early indicators of Alzheimer’s disease (Rémy et al., 2005; Gold and Budson, 2008) with semantic deficits appearing only as the disease progresses (Sánchez et al., 2017). That is, it is possible that J20s and in 52-week-old male wild-types suffered impaired episodic-like memory which led to their initial deficit in goal-directed action, but that intact semantic-like memories in all mice allowed for intact goal-directed performance on the 8-d test. Such an interpretation is, of course, highly speculative, but does nicely illustrate the intersection of memory, motor control, and action selection, as required for diagnoses of Alzheimer’s disease in humans.

Finally, the nature of the deficit observed here has some important potential translational implications. Specifically, these results suggest that additional training allowed J20 mice to overcome their impairment to demonstrate intact goal-directed action. If translatable, this could suggest that individuals with Alzheimer’s disease might be similarly impaired when initially learning how to perform a new action or when learning to perform an action in a new environment. However, with enough training, repetition, and/or instruction, they could overcome this impairment. Such behavioral interventions are reminiscent of the “dementia villages” that have begun to be built around the world (Peoples et al., 2020), that allow individuals more time and help to make decisions about, for example, crossing the road, or what they would like to purchase from the shops. Our study provides additional validation for these kinds of interventions, suggesting that they can even be modelled in mice.

Overall, we hope that the findings reported here provide an important starting point for researchers wishing to test a more comprehensive set of behavioral assays in preclinical mouse models of Alzheimer’s disease. The current study is the first, to our knowledge, that investigates how such visual and spatial memory deficits might translate into poorer cognitive control over actions. In parallel, individuals with Alzheimer’s disease will only reach a diagnosis if their memory deficits impair their ability to function independently. Therefore, we hope that these findings will replicate in distinct mouse models of Alzheimer’s disease, and to this end, we are more than happy to provide additional details on our protocols or any other aspect of our design that we may have neglected to mention here. We have also shared all of our data online at the following DOI: 10.17 605/OSF.IO/JXYC9, should researchers want to conduct their own analyses.

## Synthesis

Reviewing Editor: Christina Zelano, Northwestern University - Chicago Decisions are customarily a result of the Reviewing Editor and the peer reviewers coming together and discussing their recommendations until a consensus is reached. When revisions are invited, a fact-based synthesis statement explaining their decision and outlining what is needed to prepare a revision will be listed below. The following reviewer(s) agreed to reveal their identity: Rachel Anderson.

The reviewers and I agree that this paper is interesting and makes an important contribution to the field as it is important that mouse models of human disease are tested with more complex behavioral analyses. However the reviewers raised important concerns that need to be addressed, see below:

Reviewer 1

The paper “Goal-directed action is transiently impaired in a hAPP-J20 mouse of Alzheimer’s disease” investigates how outcome devaluation is affected in a mouse model of Alzheimer’s disease. In this paper, both male and female mice were trained to press levers for two distinct outcomes, and then tested for their preference of those outcomes following a period of satiation on one of the two rewards. 36-week old male J20 mice did not show appropriate devaluation of the reward, showing equal preference for both. The same trend was observed in 52-week old male mice, regardless of genotype. Following 4 weeks of additional lever press training, all male mice, regardless of age or genotype showed appropriate devaluation behavior. Female mice showed similar trends, with J20 female mice showing impaired devaluation initially, but intact devaluation after an additional 4 weeks of training. Microglia activation, amyloid plaques, and astrocyte activation were measured in dCA1 with migrocial activation being negatively correlated with devaluation behavior in male mice during initial testing.

Overall, I think this is a good paper. The methodology and statistical analysis are strong. That paper is well written and easy to follow. The figures are easy to read and understand. I think the findings are important in that they argue for more complex behavioral analysis of mouse models of human disease.

My main concern is that of interpretation. To me, it is clear that the findings of “transient impairment” are more indicative of a potential learning deficit yet they are not framed as such. Without further clarification of why it is a transient impairment, instead of a learning impairment (or maybe they are the same thing?) I do not understand the framing. I would appreciate further clarification on this. The rest of my concerns are largely minor and are mostly areas where I would like further clarification.

Minor concerns

Introduction:

Last paragraph on 3rd page: authors describe that outcome devaluation has been translated to humans and tested in “individuals with several different diseases and disorders”. Has this ever been tested in Alzheimer’s? If so, this information should be included. If not, a better explanation for why this is a valid test for AD would be helpful

Page 4: It is not clear to me why it was expected that J20 would show only transient impairments? Without reading Bradfield et al., 2020 the logic here is missing. And even with that understanding, why did the authors expect J20 mice to show the same transient deficits? At least a brief explanation of why this would be expected would be helpful

Materials and Methods:

I think it should be made clear in the methods section why the training and testing were different between males and females. If one reads the article as written one ends up confused by the difference. It is only until you get to the results that you understand the reasoning.

Results:

What constitutes baseline responding? This should be explained in the methods

Figures

1A should be altered to show that there are 2 days for devaluation and 4 day test

Figure 7H & I: it would be nice to see which animals were WT or J20

Discussion:

To me, it is very clear that this is a learning effect. J20 animals need more training to show goal-directed behavior. I struggle with the idea that through this lens, this would be considered a “transient impairment” but maybe more of a learning deficit. It makes sense that both J20 mice and aged mice would need more trials to learn. I feel like discussion of this aspect is treated as an afterthought, when I think the evidence highly suggests that it is the case.

If IBA1 is implicated in the impaired devaluation, I am curious as to potential explanations of why it has a “transient” effect at the 4 day test but not the 8 day test. Does microglia activation decrease with learning? Discussion of how these are tied would be appreciated.

Reviewer 2

Abstract needs grammar improvement and correction for some strange word choices.

The intro can also be improved (lots of repetitions)

Page 11: There is a lot of repetition regarding methodological considerations already explained in Materials and Methods. Detailed explanations of methodological choices should be moved to the mats and methods section and removed from the description of results.

Page 11: not sure what p=.00 means (also appear in other parts of the manuscript)

Fig.1c: Training may not have been sufficient for 52-week-old mice, which explains the lack of devaluation effect, including WT mice. Besides the motor bias that has been comprehensively analyzed, the cumulative number of presses over the 4 days of training for 52-week-old mice is significantly reduced compared to younger mice and might not be enough to develop goal-oriented behavior. This hypothesis is reinforced by the fact that all genders and genotypes are susceptible to devaluation after 8 days of training (when older mice exhibit similar press performance to younger mice, at least for WT groups).

Fig. 1c: Surprisingly, some individual J20 mice are sensitive to devaluation (as opposed to all WTs are sensitive to devaluation), but others are resistant to devaluation, resulting in a non-significant devaluation effect as than average. Do the authors have an explanation for this? This should be mentioned in the discussion.

Fig. 3b: The authors state that 52-week-old J20 mice show impaired reintegration, but the press performance of 52-week-old J20 mice was significantly lower than other cohorts at days 7 and 8. This is a major bias, it could have explained that the performance after reintegration of the 52-week-old J20 mice is lower than that of the other cohorts and does not differ significantly from the non-reintegrated group.

Page 17: There is a lot of repetition regarding methodological considerations already explained in Materials and Methods.

Fig. 4: Although they explained why, the fact that the authors chose to modify the training procedures of the female mice makes it difficult to compare with the male data. Even if, under these conditions, female J20 mice reproduced the defect of transient devaluation observed in males, this might not be the case with training similar to that of males.

Page 21: The authors should probably delete the explanation of the freezing problem altogether or move it to the mats and methods section.

As mentioned in the authors’ discussion, Alzheimer’s disease has a higher prevalence in women than in men. Thus, it is important to ensure that the primary outcome claimed by this paper, the transient devaluation effect, is also present in female mice by testing at 52 weeks.

The beginning of the discussion should be rephrased to avoid repeating the description of the results.

The impact of hippocampal neuroinflammation on devaluation sensitivity is unclear. Primarily, the authors report transient goal-directed impairment in J20 mice but show no differences in HC inflammation between genotypes. How do the authors explain this discrepancy?

## Author Response

Reviewer 1

The paper “Goal-directed action is transiently impaired in a hAPP-J20 mouse of Alzheimer’s disease” investigates how outcome devaluation is affected in a mouse model of Alzheimer’s disease. In this paper, both male and female mice were trained to press levers for two distinct outcomes, and then tested for their preference of those outcomes following a period of satiation on one of the two rewards. 36-week old male J20 mice did not show appropriate devaluation of the reward, showing equal preference for both. The same trend was observed in 52-week old male mice, regardless of genotype. Following 4 weeks of additional lever press training, all male mice, regardless of age or genotype showed appropriate devaluation behavior. Female mice showed similar trends, with J20 female mice showing impaired devaluation initially, but intact devaluation after an additional 4 weeks of training. Microglia activation, amyloid plaques, and astrocyte activation were measured in dCA1 with migrocial activation being negatively correlated with devaluation behavior in male mice during initial testing.

Overall, I think this is a good paper. The methodology and statistical analysis are strong.

That paper is well written and easy to follow. The figures are easy to read and understand. I think the findings are important in that they argue for more complex behavioral analysis of mouse models of human disease.

My main concern is that of interpretation. To me, it is clear that the findings of “transient impairment” are more indicative of a potential learning deficit yet they are not framed as such. Without further clarification of why it is a transient impairment, instead of a learning impairment (or maybe they are the same thing?) I do not understand the framing. I would appreciate further clarification on this. The rest of my concerns are largely minor and are mostly areas where I would like further clarification.

We thank the reviewer for this important point, and agree that the deficit in J20 animals is better characterised as an ‘initial’ impairment in goal-directed action that can be overcome with additional training. We had based the ‘transient’ interpretation on the previous findings that goal-directed action becomes hippocampally-independent over time, regardless of whether or not additional training is given. However, as that was not the protocol used here we cannot apply to same interpretation. Therefore, we have re-worded our terminology throughout the manuscript to reflect this characterisation.

Although the reviewer is correct that the observed impairment is consistent with learning deficit, this is a little difficult to say conclusively because the genetic manipulation is present throughout the entire experiment - including both learning and test phases.

Although unlikely, it is possible that there is a deficit in the initial expression of goal-directed action on the 4 day test that is removed by the time of the 8 day test. Moreover, during the lever press acquisition, when the ‘learning’ is occurring, there is no difference in lever press rates between wildtype and J20 mice of either sex. Therefore, although the 2 pattern of results is more consistent with a learning deficit than a performance/expression deficit, we cannot say this for certain.

In addition to removing the word ‘transient’ from the manuscript, we have added the following to the discussion:

"Nevertheless, the behavioural finding that goal-directed action was initially impaired in 36- week-old mice of both sexes parallels previous findings that inactivating the dorsal CA1 region of the hippocampus also initially impairs goal-directed action, but that such actions become hippocampally-independent with additional training or over time (Bradfield et al., 2020). The fact that both J20 and aging male mice also overcame this deficit after additional lever press training suggests that these animals were slower to learn the underlying action outcome contingencies. It is important to note, however, that this interpretation is somewhat limited by the fact that these manipulations (i.e. genotype and age) were present throughout all phases of the experiment, including acquisition and test phases, making it difficult to confidently separate whether this was an impairment in the initial learning or expression of goal-directed action.”

Minor concerns

Introduction:

Last paragraph on 3rd page: authors describe that outcome devaluation has been translated to humans and tested in “individuals with several different diseases and disorders”. Has this ever been tested in Alzheimer’s? If so, this information should be included. If not, a better explanation for why this is a valid test for AD would be helpful

Outcome devaluation has not been tested in Alzheimer’s, although based on the preclinical work we present here we would suggest that it would be a good idea to do so. We have added the following passage and citations to the introduction (Page 3) to explain why this is a valid test for AD:

"Goal-directed action control is crucial to independent functioning and its impairment in individuals with Alzheimer’s can be devastating as it prevents individuals from reaching their goals and from making effective decisions (Brown & Pluck, 2000; Satler et al., 2017).”

And on Pages 3-4:

"Goal-directed action is defined as action motivated by a) the current value of the outcome and b) the contingency between the action and the outcome (Balleine & Dickinson, 1998).

Outcome devaluation provides a test of these criteria. The organism is typically trained to perform two actions (e.g. left and right lever press) for two outcomes (e.g. pellets and sucrose), the value of one of which is subsequently devalued, often by being fed to satiety or paired with illness. When tested in the absence of the outcomes, the organism that selects the action associated with the still valued outcome is acting under goal-directed 3 control because they are responding in accordance with both the outcome’s value (value criterion) and their memory of which action earned which outcome (contingency criterion).” Page 4: It is not clear to me why it was expected that J20 would show only transient impairments? Without reading Bradfield et al., 2020 the logic here is missing. And even with that understanding, why did the authors expect J20 mice to show the same transient deficits? At least a brief explanation of why this would be expected would be helpful

We have re-worded this passage to now read:

"This is of interest because we recently demonstrated that inactivating this same brain region caused an initial deficit in devaluation performance that could be overcome with additional training (Bradfield et al., 2020). Therefore, we hypothesised that the damage suffered to the dorsal CA1 in J20 mice would likewise cause an initial impairment in devaluation that could be overcome with additional training.”

Materials and Methods:

I think it should be made clear in the methods section why the training and testing were different between males and females. If one reads the article as written one ends up confused by the difference. It is only until you get to the results that you understand the reasoning.

We have moved the following passage from the results to under the ‘subjects’ subheading in the methods section:

"We have reported results for female mice separately below because slightly different experimental parameters were used for male and females due to female mice exhibiting substantially lower lever press rates.”

Results:

What constitutes baseline responding? This should be explained in the methods

We have added the following passage under the ‘Experimental design and statistical analysis’ subheading in the methods section on page 11:

"For test data, both the raw scores and scores as a percentage of baseline responding are reported. Baseline was defined as the lever press rates per minute on each lever, averaged across the two days of training immediately prior to test (i.e. for the 4 day test this was days 3-4, for the 8 day test this was days 7-8). “ 4 Figures 1A should be altered to show that there are 2 days for devaluation and 4 day test

We thank the reviewer for this point, and although we tried to accommodate it in Figure 1A, we ultimately decided that it would make the figure more confusing, particularly given the use of ‘4 Day’ and ‘8 Day’ to differentiate the tests days from each other based on the number of days of lever press training that occurred prior to each test. We did, however, alter the devaluation phase to show that both outcome 1 and outcome 2 were prefed to satiety during this phase. We hope that this, along with the information included in the text about how tests were conducted, is sufficient and note that note that similar or identical table designs have been used in many previous publications (e.g. DOIs: 10.1016/j.neuron.2015.10.044; 10.1038/s41593-020-0693-8; 10.1523/JNEUROSCI.3860-16.2017) Figure 7H & I: it would be nice to see which animals were WT or J20 Great suggestion, thank you. This has been altered.

Discussion:

To me, it is very clear that this is a learning effect. J20 animals need more training to show goal-directed behavior. I struggle with the idea that through this lens, this would be considered a “transient impairment” but maybe more of a learning deficit. It makes sense that both J20 mice and aged mice would need more trials to learn. I feel like discussion of this aspect is treated as an afterthought, when I think the evidence highly suggests that it is the case.

Thank you again for this important point and we agree. As well as removing reference to

‘transient’ deficits and replacing it with reference to an ‘initial’ deficit in goal-directed action, we have added the text mentioned above to the discussion (Page 29) to address this directly.

If IBA1 is implicated in the impaired devaluation, I am curious as to potential explanations of why it has a “transient” effect at the 4 day test but not the 8 day test.

Does microglia activation decrease with learning? Discussion of how these are tied would be appreciated.

This is a great point, and it is worth noting that the microglial expression is quantified at the end of the experiment so it is the same microglial expression that we are either correlating with the devaluation ratio from the 4 day test or the 8 day test. That is, the quantification of the microglial expression does not change, only the devaluation score does. The fact that it only correlates with performance on the 4 day test and not the 8 5 day test we think is reflective of the fact that neuroinflammation in the dorsal hippocampus only affects initial goal-directed action/performance on the 4 day test, whereas performance on the 8 day test is hippocampally-independent. We added the following new text (in red) to the discussion on page 30 to try and make this clearer:

"To our knowledge, the dorsal CA1 is the only brain region upon which goal-directed action is initially dependent, but becomes independent of after multiple days of training. By contrast, lesions and/or inactivations of alternate brain regions, such as posterior dorsomedial striatum (Yin et al., 2005), nucleus accumbens core (Corbit et al., 2001), prelimbic cortex (Corbit and Balleine, 2003), basolateral amygdala (Ostlund and Balleine, 2008), medial orbitofrontal cortex (Bradfield et al., 2015; 2018), and mediodorsal thalamus (Corbit et al., 2003), have all produced sustained impairments in goal-directed action after multiple days of lever press training (although note that devaluation is sometimes intact if the manipulation occurs post-training). This fact, coupled with the negative correlation between dorsal CA1 microglial expression and initial devaluation performance strongly suggests that the goal-directed deficits observed here were also a result of damage to this region. It is possible that future studies will find neuroinflammation in the other brain regions mentioned to be associated with devaluation performance on tests after 8 or more days of lever press training, in line with the more sustained roles these regions play in goal-directed action.”

Reviewer 2

Abstract needs grammar improvement and correction for some strange word choices.

The intro can also be improved (lots of repetitions) In line with the suggestions of reviewer #1, throughout the manuscript we have removed reference to ‘transient’ deficits in goal-directed action and replaced them with reference to ‘initial’ deficits in such actions. We hope this is clearer. We have also read through the abstract and introduction removed several clunky words and passages, and tried to add clarity through several sessions. We hope that this addresses the reviewer’s concerns, but if there are any specific word choices or passages that the reviewer continues to find strange we are happy to remove and/or reword the relevant sections. Page 11: There is a lot of repetition regarding methodological considerations already explained in Materials and Methods. Detailed explanations of methodological choices should be moved to the mats and methods section and removed from the description of results.

We have removed several repetitious methods from the results section and moved some information to the methods section. We hope this is sufficient but again, if there are any repetitions remaining we are happy to remove those also. 6 Page 11: not sure what p=.00 means (also appear in other parts of the manuscript)

We apologise for the confusion. In rounding our p values down to two decimal places, we sometimes rounded these values down to zero. However, we have replaced all p = .00 with either a more specific p value to more decimal places, or with p <.00001 Fig.1c: Training may not have been sufficient for 52-week-old mice, which explains the lack of devaluation effect, including WT mice. Besides the motor bias that has been comprehensively analyzed, the cumulative number of presses over the 4 days of training for 52-week-old mice is significantly reduced compared to younger mice and might not be enough to develop goal-oriented behavior. This hypothesis is reinforced by the fact that all genders and genotypes are susceptible to devaluation after 8 days of training (when older mice exhibit similar press performance to younger mice, at least for WT groups).

Similar to reviewer #1, this reviewer is suggesting that the deficit is perhaps in learning rather than in goal-directed action per se. We have altered the language throughout the manuscript to reflect this. Fig. 1c: Surprisingly, some individual J20 mice are sensitive to devaluation (as opposed to all WTs are sensitive to devaluation), but others are resistant to devaluation, resulting in a non-significant devaluation effect as than average. Do the authors have an explanation for this? This should be mentioned in the discussion.

This is a really interesting point, and something we have thought a lot about. The individual patterns of responding from Figure 1C in particular are very interesting in a way that is not captured by the overall statistical analysis, emphasising why it is so important to show individual data points on graphs.

Our interpretation of these patterns is that, just as there is a great deal of variability in the human population in terms of cognitive capacity, so too is there variability in the cognitive capacity of individual mice. Moreover, this variability often occurs in spite of individuals exhibiting neuropathology normally associated with Alzheimer’s disease (i.e. ‘cognitively normal’ individuals who, on post-mortem, are found to express amyloid plaques and neurofibrillary tangles usually indicative of an Alzheimer’s diagnosis). This is now reflected in the following passage added to the discussion on page 28:

"Interestingly, several of the individual mice from the ‘impaired’ groups demonstrated intact devaluation (see Figures 1C and 3B), suggesting that some of these mice were able to exhibit goal-directed action. We interpret this as reflective of a variety of cognitive abilities in these mice despite their genotype and/or age, and note that such variability has been observed in older humans, as well as humans with Alzheimer’s disease (Hedden et al., 2013).” 7 Fig. 3b: The authors state that 52-week-old J20 mice show impaired reintegration, but the press performance of 52-week-old J20 mice was significantly lower than other cohorts at days 7 and 8. This is a major bias, it could have explained that the performance after reintegration of the 52-week-old J20 mice is lower than that of the other cohorts and does not differ significantly from the non-reintegrated group.

There is a little bit of confusion here. The test is of outcome-selective reinstatement, not reintegration, and the comparison is within-subjects (i.e. pressing on the reinstated vs. the non-reinstated lever) for each genotype, with genotype being the between-subject factor. Therefore, there is no ‘non-reintegrated group’. Moreover, none of our reported statistics suggest that the 52 week old wildtypes differed in their responding from the 52-week-old J20s during lever press training, as there was no main effect of genotype and no interactions with genotype. The only significant differences we reported in lever press training were due to age.

Nevertheless, as a result of the reviewer’s comment, we did look at the simple effects for days 7 and days 8 alone, and although there is no significant simple effect on day 7, F (1,35) = 2.595, p = .116, there is (just!) a significant simple effect on day 8, F (1,35) = 4.138, p = .05, suggesting that 52-week-old wildtypes pressed significantly more than J20s on day 8 only. As a result, we investigated whether transforming the reinstatement data as a percentage of baseline responding would ‘rescue’ the deficit in 52 week old J20s and it did not. The graph is shown in Figure 3B, and the statistical analyses reported on page 17. To be consistent, we also transformed the performance of 36-week-old males on this test, shown in Figure 3E, and the performance of 36-week-old females, shown in Figure 6B. The relevant statistics are now reported in text on Page 17 for males, and Pages 21-22 for females. Page 17: There is a lot of repetition regarding methodological considerations already explained in Materials and Methods. We have removed several repetitive passages. Fig. 4: Although they explained why, the fact that the authors chose to modify the training procedures of the female mice makes it difficult to compare with the male data. Even if, under these conditions, female J20 mice reproduced the defect of transient devaluation observed in males, this might not be the case with training similar to that of males.

This is a great point, and is exactly why we were careful not to directly compare the performance of males to females. Rather, we decided to report the results for each separately. As females have smaller bodies and eat less, it is our suspicion that this is the reason for the females not pressing the lever at rates equivalent to males and why we 8 needed to modify parameters. We would further like to note that the parameters of any experiment are somewhat arbitrary, but because of the historical bias against using female animals in studies, the ‘default’ parameters for most experiments tend to be those that have targeted males.

Based on our results, what we can say with a strong degree of certainty is that, using the particular parameters we used, at 36 weeks of age both male and female wildtypes are capable of showing intact outcome devaluation performance and goal-directed action, whereas J20s are not. It is also worth noting that to reach this conclusion about goal-directed action, we relied on the relative responding on the valued and devalued levers, whereas the rate of responding is less important. Page 21: The authors should probably delete the explanation of the freezing problem altogether or move it to the mats and methods section.

We have moved this to materials and methods, page 8.

As mentioned in the authors’ discussion, Alzheimer’s disease has a higher prevalence in women than in men. Thus, it is important to ensure that the primary outcome claimed by this paper, the transient devaluation effect, is also present in female mice by testing at 52 weeks.

As the reviewer mentions, the study would ideally contain a cohort of 52-week-old females, particularly given the increased prevalence of Alzheimer’s in human females relative to males. It was a great disappointment to us that we did not have this cohort available. Unfortunately, because the inclusion of such a cohort would involve ageing a sufficient number of females to 52 weeks of age, and we cannot guarantee litters of a particular size, the inclusion of such a cohort is beyond the realms of feasibility for the current manuscript.

The beginning of the discussion should be rephrased to avoid repeating the description of the results.

We thank the reviewer for the suggestion but respectfully disagree. We have kept the result description in line with the long-established convention in behavioural neuroscience for the first paragraph of the discussion to recap the main findings of the paper
